# Hybrid Boolean gates show that Cas12c controls transcription activation effectively in the yeast *S. cerevisiae*

**DOI:** 10.3389/fbioe.2023.1267174

**Published:** 2023-09-12

**Authors:** Yifan Liu, Huanhuan Ge, Mario Andrea Marchisio

**Affiliations:** School of Pharmaceutical Science and Engineering, Tianjin University, Tianjin, China

**Keywords:** CRISPR-Cas12c, hybrid Boolean gates, riboswitches, *saccharomyces cerevisiae*, anti-CRISPR

## Abstract

Among CRISPR-Cas systems, type V CRISPR-Cas12c is of significant interest because Cas12c recognizes a very simple PAM (TN) and has the ability to silence gene expression without cleaving the DNA. We studied how new transcription factors for the yeast *Saccharomyces cerevisiae* can be built on Cas12c. We found that, upon fusion to a strong activation domain, Cas12c is an efficient activator. Its functionality was proved as a component of hybrid Boolean gates, i.e., logic circuits that mix transcriptional and translational control (the latter reached via tetracycline-responsive riboswitches). Moreover, Cas12c activity can be strongly inhibited by the anti-CRISPR AcrVA1 protein. Thus, Cas12c has the potential to be a new tool to control the activation of gene expression within yeast synthetic gene circuits.

## Introduction

CRISPR-Cas is an RNA-based immune system in bacteria and archaea (Barrangou et al., 2007; Marraffini and Sontheimer, 2010). CRISPR-associated effector nucleases (Cas) pair with CRISPR RNA (crRNA) molecules to bind and cleave exogenous nucleic acid sequences. In this way, CRISPR-Cas protects cells against phages, plasmids, and other mobile genetic elements (Horvath and Barrangou, 2010). CRISPR-Cas systems are divided into two classes and six types. Class 2, which gathers type II, V, and VI, necessitates of a single Cas protein to carry out DNA (or RNA) cleavage. Thus, Cas9 (type II) and Cas12a (type V) have been widely used in gene editing (Karvelis et al., 2017; Yan et al., 2019). Type V Cas12c stands out in this scenario because of its unique features. Even though Cas12c possesses a RuvC nuclease domain similar to that of other Cas proteins, Cas12c does not show any DNase activity, whereas it can process its own pre-crRNA into mature crRNA (Huang et al., 2022). The mechanisms through which Cas12c make the cells resist phage infection are yet to be elucidated. Nevertheless, Cas12c represents a favorable template to engineer transcription factors since no DNase domain shall be mutated.

RNA structures such as riboswitches carry out a post-transcriptional control on gene expression and, to this aim, they have been adopted in both prokaryotic and eukaryotic genetic circuits (Benenson, 2012; Ge and Marchisio, 2021). Riboswitches are made of an aptamer (the sensor domain), where chemicals bind and induce a change in the expression platform (the regulatory domain), which has repercussions on mRNA translation. Riboswitches are relatively short and, in nature, are found in the 5′ UTR (untranslated region) of (mainly) bacterial genes (Mellin and Cossart, 2015; McCown et al., 2017). Upon binding a chemical, most natural riboswitches prevent translation from starting (Wunnicke et al., 2011). In this way, they mimic the NOT Boolean function that returns ‘1’ (gene expression) as an output in the absence (‘0’) of its only input (the chemical).

Logic gates are the fundamental building blocks of digital circuits that permit to realize biosensors (Marchisio, 2014). In biology, a single transcription unit (TU, i.e., the DNA sequence promoter-gene-terminator) can reproduce a multi-input logic function by exploiting mechanisms for the control of transcription and/or translation (Marchisio and Stelling, 2011). Biological Boolean gates that combine transcription and translation regulation to mimic a logic function are referred to as hybrid gates (Marchisio and Stelling, 2011). For instance, a promoter that host operators (i.e., the binding sites of transcription factors) upstream of the TSS (transcription start site) and encodes for at least one riboswitch along the 5’ UTR is a generic, simple design for a hybrid gate (see Supplementary Figure S1). Synthetic hybrid gates have been successfully realized in bacteria and mammalian cells (Leisner et al., 2010; Morra et al., 2016).

In this work, we present a first characterization of Cas12c as a transcription factor in the yeast *Saccharomyces cerevisiae*. Similar to other type V Cas proteins, Cas12c appears more effective as an activator rather than a repressor. Moreover, its activity can be controlled by AcrVA1, whereas AcrVA4 and AcrVA5 (Yu and Marchisio, 2020) have no effect on it. We tested Cas12c behavior as a signal carrier in synthetic gene hybrid gates—where translation was downregulated by up-to-two riboswitches responding to tetracycline (Kötter et al., 2009). For a comparison, we implemented several different hybrid gates that made use of transcription factors based on either type V denAsCas12a (Kleinstiver et al., 2019; Yu and Marchisio, 2021) or LexA-HBD (hER) (i.e., the bacterial protein LexA fused to the hormone-binding domain of the human estrogen receptor) (Louvion et al., 1993; Zhou et al., 2022). Alternatively, we also employed promoters regulated by internal pathways (e.g., the *GAL1* promoter). Overall, we identified the best designs for riboswitch-containing hybrid gates and proved that Cas12c worked efficiently as an activator inside *Saccharomyces cerevisiae* synthetic gene circuits.

## Results and discussion

### Cas12c regulates transcription in *Saccharomyces cerevisiae* upon fusion to an effector domain

In order to establish if the bare Cas12c could be a repressor in yeast cells, we fused it, initially, to two nuclear localization sequences (NLSs) only and put it under the control of the strong constitutive *GPD* promoter (pGPD). The yeast enhanced green fluorescent protein (yEGFP (Sheff and Thorn, 2004)) was chosen as a target and expressed under the *TEF2* promoter (pTEF2, 44% of the strength of pGPD—see Table SP). Cas12c recognizes a short protospacer adjacent motif (PAM, 5′-TN) (Huang et al., 2022; Zhang et al., 2022) slightly different from that of Cas12a (5′-TTTV, V: NOT T) (Zetsche et al., 2015). The crRNA was expressed via RNA polymerase III-type elements (the *SNR52* promoter—pSNR52, and the *SUP4* terminator—SUP4t (DiCarlo et al., 2013)). As a particular feature of Cas12c, the length of the spacer in the crRNA is fixed to 17 nt (Huang et al., 2022). In a previous work from our lab (Yu and Marchisio, 2021), we pointed out that dCas12a exerted, in yeast cells, the highest fluorescence inhibition by targeting the mid region of *yEGFP*. Therefore, the crRNA in our first circuit (Circuit S1, see Supplementary Figure S2A) contained spacer1 that bound the middle of the *yEGFP* gene on the antisense strand. However, this design did not lead to a significant fluorescence reduction (see Supplementary Figure S3B; Supplementary Table S1). Therefore, we designed more spacers to target pTEF2 on both the sense (bS—spacer3 and spacer4) and the antisense strand (bA—spacer2 (Farzadfard et al., 2013), see Supplementary Figure S2C). In term of OFF/ON ratio, the best value was achieved by expressing spacer3 and spacer4 together. However, fluorescence decreased by 40% only (see Supplementary Figure S2B).

To improve the action of Cas12c as a repressor, we fused it to the mammalian repression domain Mxi1 that was reported to interact with the histone deacetylase Sin3 homolog in yeast (Gilbert et al., 2013). The chimeric protein Cas12c-Mxi1 made a complex with the same crRNAs as in the previous test (Circuit 1, see Supplementary Figure S3A). Repression efficiency increased, even though the best results was achieved by means of a single crRNA, which contained spacer4 (63% of fluorescence reduction—see Supplementary Figure S3B; Supplementary Table S2). Thus, to inhibit transcription up to a reasonable level, Cas12c shall be fused to a repression domain and target a sequence along a promoter.

Cas12c was, then, turned into an activator by fusing it to the strong VPR (VP64-P65-Rta) activation domain (D et al., 2020). We tested the functionality of Cas12c-VPR on a weak synthetic promoter made of a short variant of the core yeast *CYC1* promoter (trunc_pCYC1core: the 5’ UTR is only 24 nt long instead of 71 nt and the TATA box starting at position −106 has been removed (D et al., 2020; Zhang and Marchisio, 2022)) preceded by three copies of lexOpR, i.e., the right half of the full lex2Op (Wertman and Mount, 1985) (see Circuit 2 in Supplementary Figure S3C). By binding a 17-nt-long portion of lexOpR either on the antisense (spacer5) or the sense (spacer6) strand, Cas12c-VPR managed to enhance the expression of green fluorescence up to 2.86-fold with respect to the control circuit. The activator did not show any preferential DNA strand to enhance transcription (Supplementary Figure S3D; Supplementary Table S3). It should be noted that the activator dCas9-VPR—characterized deeply in another work by our lab (Zhang and Marchisio, 2022) had a lower effect on the same target promoter (2.05-fold increase in green fluorescence) that was improved up to 2.87-fold (roughly the same as Cas12c-VPR) by substituting VPR with the smaller VP64 activation domain.

### Hybrid Boolean gates based on a tetracycline-responsive riboswitch

#### The simplest design: a single transcription unit.

The tetracycline-responsive riboswitch has been shown to downregulate gene expression effectively in *S. cerevisiae* (Suess et al., 2003). Thus, it appeared to be the best component to regulate translation within a hybrid gate. We considered two different configurations of the tetracycline-responsive riboswitch: tc1 (single copy) and tc2 (tandem) (Kötter et al., 2009). Initially, they were inserted into the 5′UTR of chemical-regulated promoters (i.e., yeast promoters responding to internal pathways that are triggered by chemicals) that drove the expression of the green fluorescent protein. This is the simplest design of a hybrid gate since it demands a single TU to process two different input signals. The promoter choice fell on *GAL1* (pGAL1, induced by galactose), *CUP1* (pCUP1, induced by copper), and *MET25* (pMET25, repressed by methionine) promoter (Kusen et al., 2017; Møller et al., 2017; Wu et al., 2023). pGAL1 and pCUP1 alone mimic a YES (or buffer) gate (i.e., the output is ‘1’ when the input is ‘1’ too), whereas pMET25 and tc1/tc2 are NOT gates (i.e., the output is ‘1’ when the input is ‘0’). The combination of pGAL1/pCUP1 with the tetracycline-responsive riboswitch shall behave as an N-IMPLY gate (i.e., fluorescence is expressed only in the presence of galactose/copper and the absence of tetracycline—see Supplementary Figure S4A–E). In contrast, pMET25 in conjunction with tc1/tc2 would mimic a NOR gate (i.e., a fluorescence signal is possible only in the absence of both methionine and tetracycline—see Supplementary Figure S4F, G, S5). In order to claim that a Boolean gate work properly, the 0s and 1s output signal shall be significantly different in statistical terms and well-separated, which corresponds to the condition: $\rho =\frac{\min\left(1\right)}{\mathrm{MAX}\left(0\right)}\approx 2$ (Marchisio, 2014; Abraha and Marchisio, 2022).

Every gate was constructed in two variants, one hosting tc1, the other tc2. Out of six gates, five showed the correct logic behavior (see Supplementary Figure S4). In general, gates hosting tc2 gave higher ρ -values than those containing tc1 (see Supplementary Table S4). This trend was evident with galactose and methionine as inputs, whereas no big difference was observed between the two N-IMPLY gates sensing copper and tetracycline. The only gate that failed to reproduce its truth table was the NOR gate with a single riboswitch. Here, tetracycline alone did not repress fluorescence expression (see Supplementary Figure S5).

#### Two-gene Boolean gates sensing ß-estradiol and tetracycline

In order to study how the performance of hybrid Boolean gates changes with circuit complexity, we designed two-gene N-IMPLY gates responding to β-estradiol and tetracycline. The promoter upstream of the *yEGFP* gene was activated by the chimeric protein made of the bacterial LexA (working as a DNA-binding domain), the hormone-binding domain of the human estrogen receptor (HDB(hER)—the docking site for β-estradiol), and an activator domain (AD) such as the strong VP64 (from the Herpes simplex virus) (Triezenberg et al., 1988) or the weaker B42, found in *Escherichia coli* (Ma and Ptashne, 1987). We expressed the chimeric activator via pGPD or the constitutive synthetic promoter DEG1t-pCYC1noTATA (Song et al., 2016) (20% as strong as pGPD, see Table SP), whereas the yEGFP was placed downstream of a minimal *CYC1* promoter (i.e., without the two UASs and the most distant TATA box from the TSS (Hahn et al., 1985)) preceded by a single or three copies of the full lex2Op and extended, along the 5’ UTR, with either tc1 or tc2. The circuit behaves as an N-IMPLY gate because β-estradiol leads to transcription activation (whereas tetracycline inhibits translation). In the absence of the hormone, HBD (hER) is bound by the heat shock protein 90 (Hps90) such that the activator is kept into the cytoplasm. In contrast, β-estradiol, when present in the cell solution, replaces Hsp90 at the HBD (hER) and allows LexA-HBD (hER)-AD to get into the nucleus (McIsaac et al., 2011) and bind the DNA at the lex2Op sites.

**FIGURE 1:**
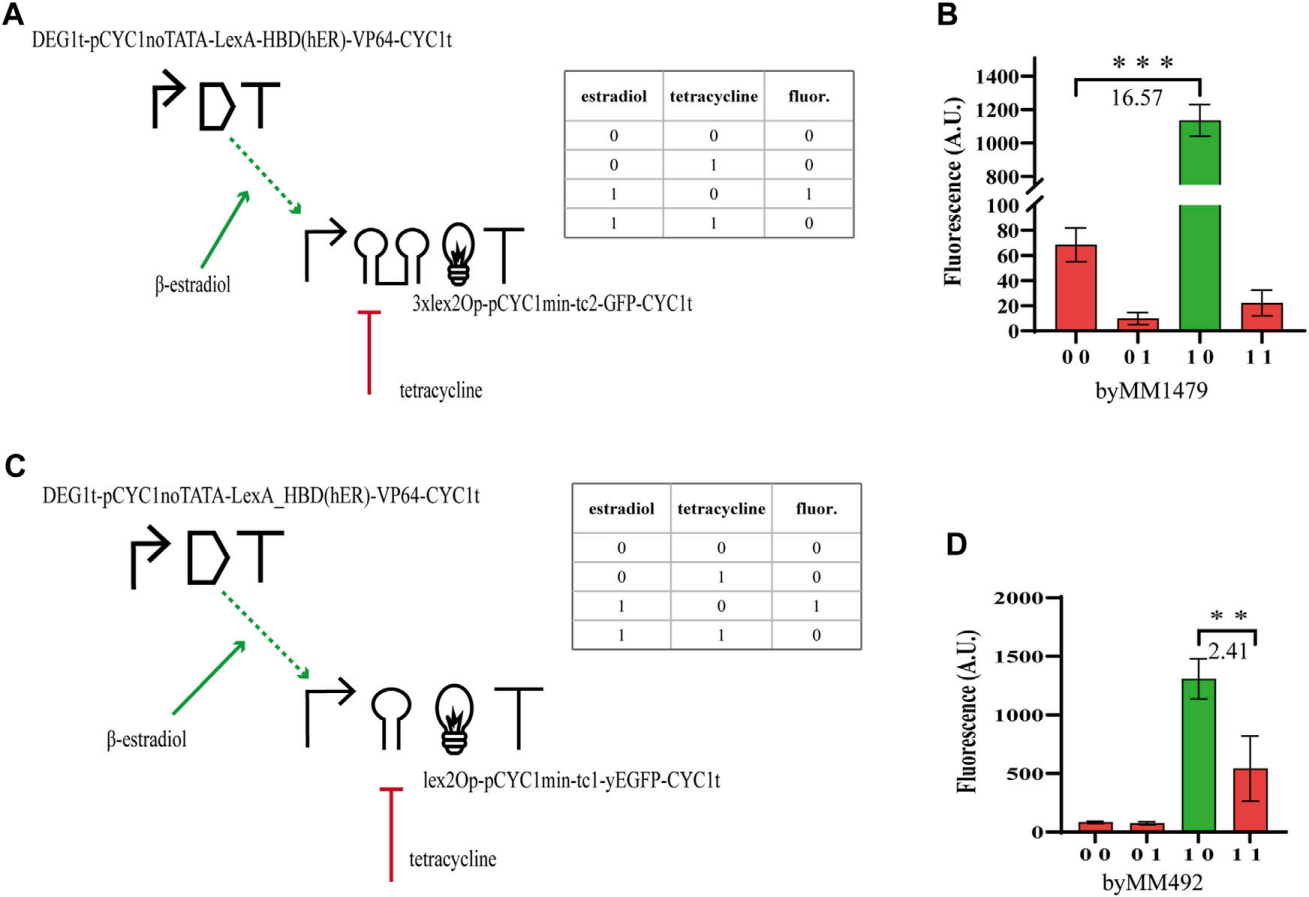
N-IMPLY gates responding to tetracycline and β-estradiol. **(A, B)** The best design requires to express LexA-HBD (hER)-VP64 under the synthetic, moderately strong, promoter DEG1t-pCYC1noTATA. Moreover, 3xlex2Op shall be present in the activated promoter together with tc2. **(C, D)** By replacing 3xlex2Op with a single lex2Op and tc2 with tc1, the ρ value undergoes a 6.9-fold decrease. The ‘1’ concentrations of the inputs are 1,000 nM β-estradiol and 130µM tetracycline. The numbers on the bar charts are the ρ values (**, *p*-value <0.01; ***, *p*-value <0.001; two-sided Welch’s t-test).

The best circuit design, which returned ρ = 16.57 (see Figures 1A, B), demanded to use (and express moderately) VP64 as an activation domain. Moreover, the activated promoter hosted the 3xlex2Op cassette and tc2 along the 5’ UTR. A similar configuration where a single lex2Op and tc1 were used reached ρ = 2.41 (see Figures 1C, D). We did not manage to improve the performance of the latter circuit with a double integration of the TU containing *yEGFP*, nor the usage of pGPD and LexA-HDB(hER)-B42 had a beneficial effect on the ρ value (see Supplementary Figure S6; Supplementary Table S5).

By making several changes on the N-IMPLY design, we engineered NOR gates responding to these two chemicals. The chimeric transcription factor was turned into a repressor by removing the AD. Moreover, LexA-HDB(hER) was expressed under pTEF2. The target, now repressed, promoter was still based on the minimal *CYC1* promoter previously used. However, the full lex2Ops upstream of the two TATA boxes were removed and a short lexOpR was placed between the TATA box starting at position −52 and the TSS. The new promoter, referred to as pCYC1min-lexOpR, was quite weak (∼250 A.U. of fluorescence in the absence of any chemical). Hence, we had to increase its strength in order to see the repression effects on the yEGFP level clearly. To this aim, we added a 40-nt-long upstream activating sequence from the *GPD* promoter (UAS(GPD-40)) 150 nt upstream of pCYC1min-lexOpR (Feng and Marchisio, 2021). Our final repressed promoter corresponded to the sequence UAS(GPD-40)-150nt (pSV40)-pCYC1min-lexOpR (i.e., the 150 nt that separated UAS(GPD-40) from pCYC1min-lexOpR were taken from the *SV40* viral promoter (Feng and Marchisio, 2021)). NOR gates hosting tc1 failed to reach a ρ value around 2 (see Supplementary Figure S7; Supplementary Table S6), whereas the variants with tc2 arrived at ρ = 2.25 (see Supplementary Figure S8; Supplementary Table S6). The latter showed that LexA-HBD (hER) could not repressed transcription strongly by binding lexOpR. The circuit performance might be increased by using the full lex2Op instead. However, other changes to the promoter sequence would be necessary to avoid, again, a too low basal transcription initiation rate.

#### Hybrid Boolean gates hosting type V CRISPR-(d)Cas12 systems

**FIGURE 2:**
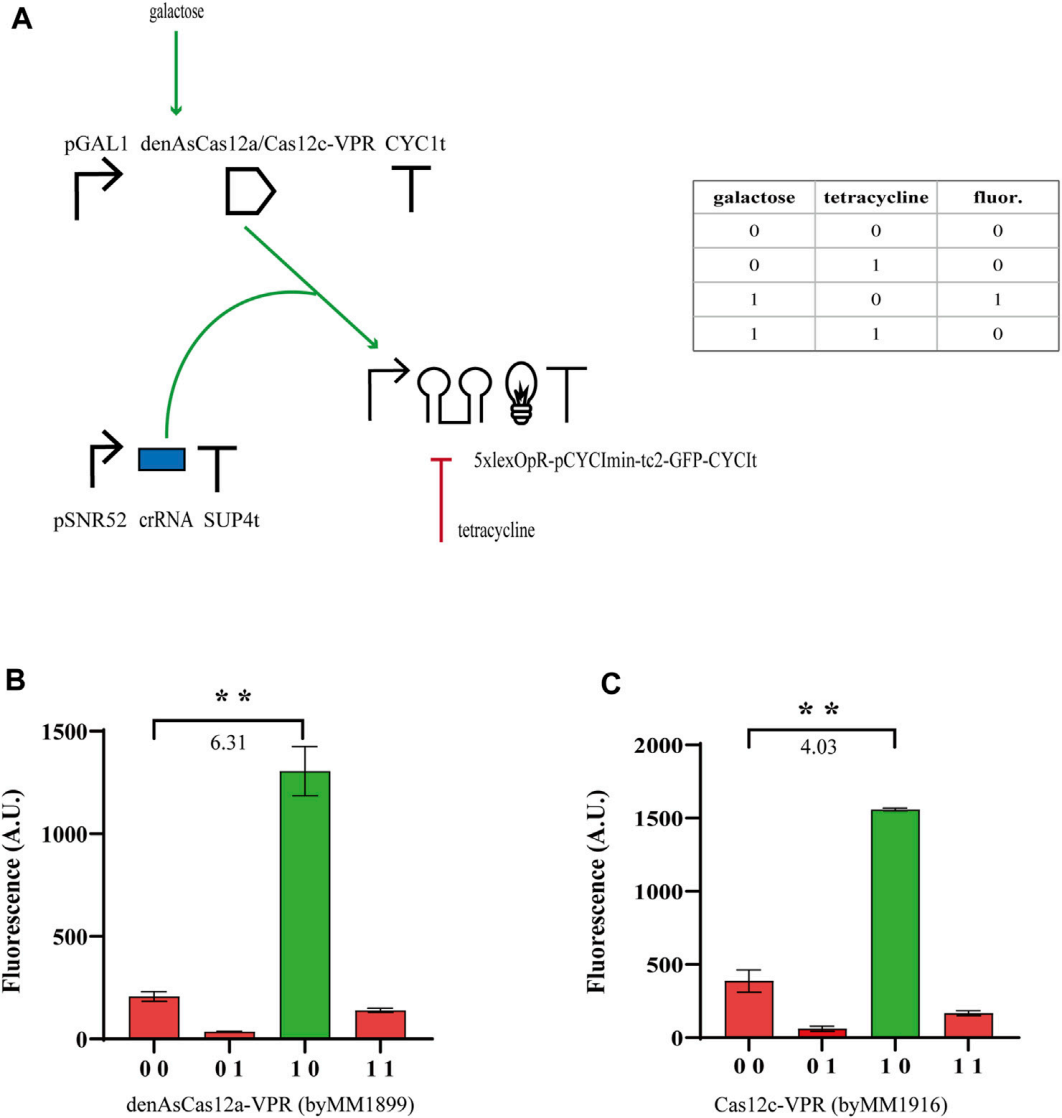
N-IMPLY gates hosting type V CRISPR-Cas systems and tc2. **(A)** Circuit scheme. (d)Cas12c protein synthesis is induced by galactose (2% in SDC solution). Tetracycline is delivered at a concentration of 130 µM tetracycline. **(B, C)** Results from denAsCas12a- and Cas12c-based gates, respectively. The numbers on the bar charts are the ρ values (**, *p*-value <0.01; two-sided Welch’s t-test).

From our previous experiments, we concluded that tc2 guaranteed, generally, higher performance than tc1 and N-IMPLY gates could reach ρ -values far larger than those obtained by NOR gates. In order to implement hybrid gates based on type V CRISPR-Cas systems, we started from denAsCa12a, on which we already built effective transcription factors (Yu and Marchisio, 2021). We fused it to the strong VPR activation domain and control its expression via pGAL1. The yEGFP was placed downstream of a synthetic activated promoter that was made of 5xlexOpR in front of the pCYC1min sequence. tc2 only was included in the 5’ UTR. The crRNA contained a 20-nt-long spacer binding lexOpR just downstream of the PAM sequence (see Figure 2A). The same design was then used to test the performance of Cas12c-VPR. In this circuit, however, the spacer was only 17 nt long (see above). Both Cas12 proteins (fused to VPR) led to well-working N-IMPLY gates. In terms of ρ -value, denAsCas12a ( ρ = 6.31) appeared more performant than Cas12c ( ρ = 4.03—see Figures 2B, C; Supplementary Table S7).

**FIGURE 3:**
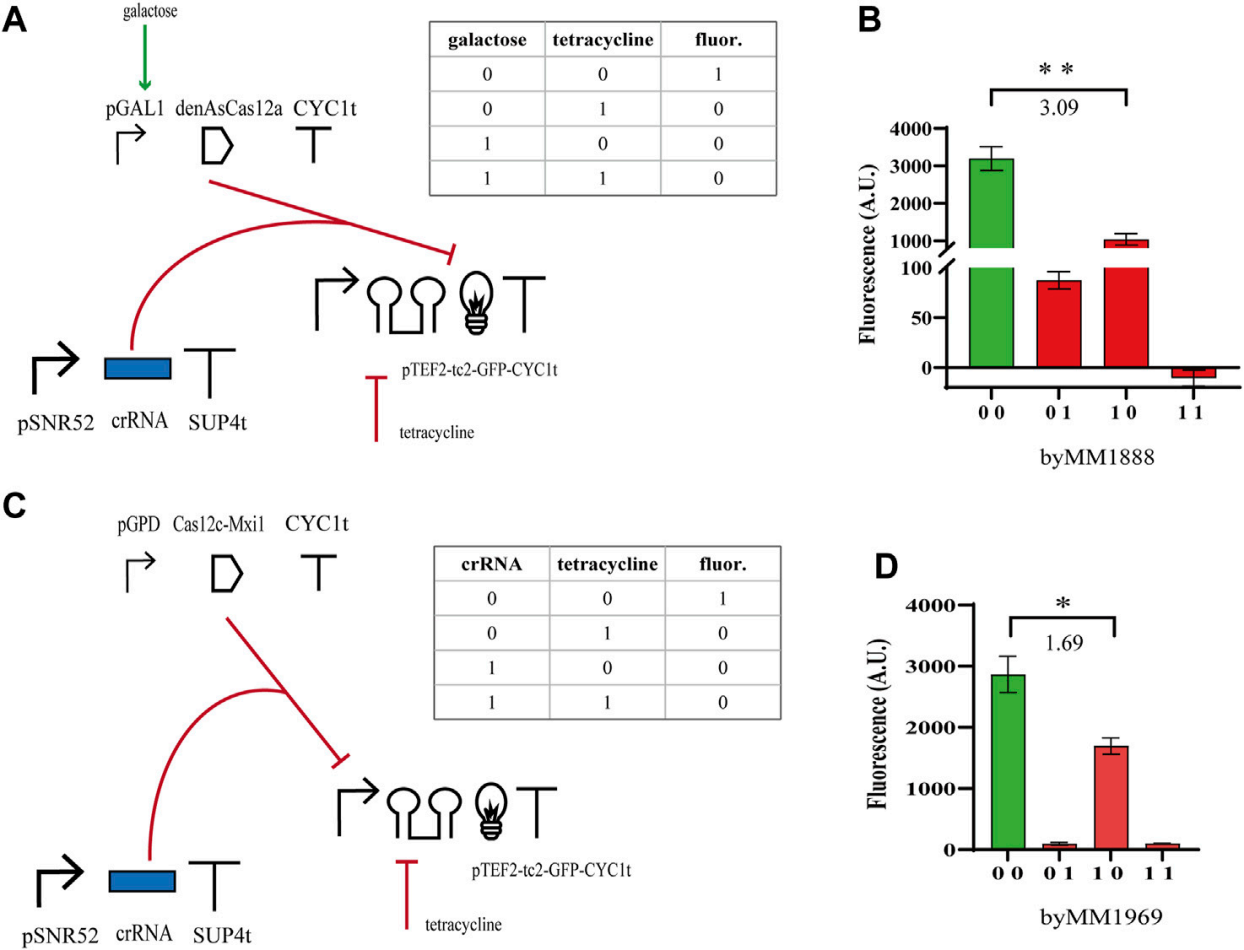
NOR gates hosting type V CRISPR-Cas systems. **(A, B)** Scheme of the NOR gate using denAsCas12a as a repressor and fluorescence levels corresponding to the four entries of the truth table. As in the previous circuits, galactose was delivered as a SDC solution (2%) and tetracycline at 130 µM. **(C, D)** Diagram of the NOR gate hosting Cas12c-Mxi1 and the corresponding output signals. Here, we considered as an input, beside tetracycline, the crRNA molecules targeting pTEF2. Therefore, the truth-table entries ‘00’ and ‘01’ correspond to a two-gene subcircuit where the crRNA expression-cassette has not been integrated into the yeast cells. The numbers on the bar charts are the ρ values (*, *p*-value <0.05; **, *p*-value <0.01; two-sided Welch’s t-test).

We moved, then, to the construction of NOR gates based on the same type V CRISPR-Cas systems. We expressed either the bare denAsCas12a (which targeted the *yEGFP* gene from position 452 to 472 with respect to the START codon) or Cas12c-Mxi1 binding pTEF2 that was placed upstream of *yEGFP*. Furthermore, denAsCas12a was expressed under the inducible *GAL1* promoter, whereas Cas12c-Mxi1 was constitutively produced by pGPD. Both gates pointed out that the effect of the repressor protein on transcription were less evident than those of tetracycline on translation. Nevertheless, the NOR gate hosting denAsCas12a returned a ρ -value slightly over than 3, whereas the NOR gate employing Cas12c-Mxi1 did not perform in a satisfactory way ( ρ = 1.69—see Figure 3; Supplementary Table S8). Taken together, these last results confirmed that denAsCas12a works remarkably well as a transcription factor in *S. cerevisiae*, whereas Cas12c can be easily turned into an effective activator only.

#### AcrVA1 inhibits Cas12c

**FIGURE 4:**
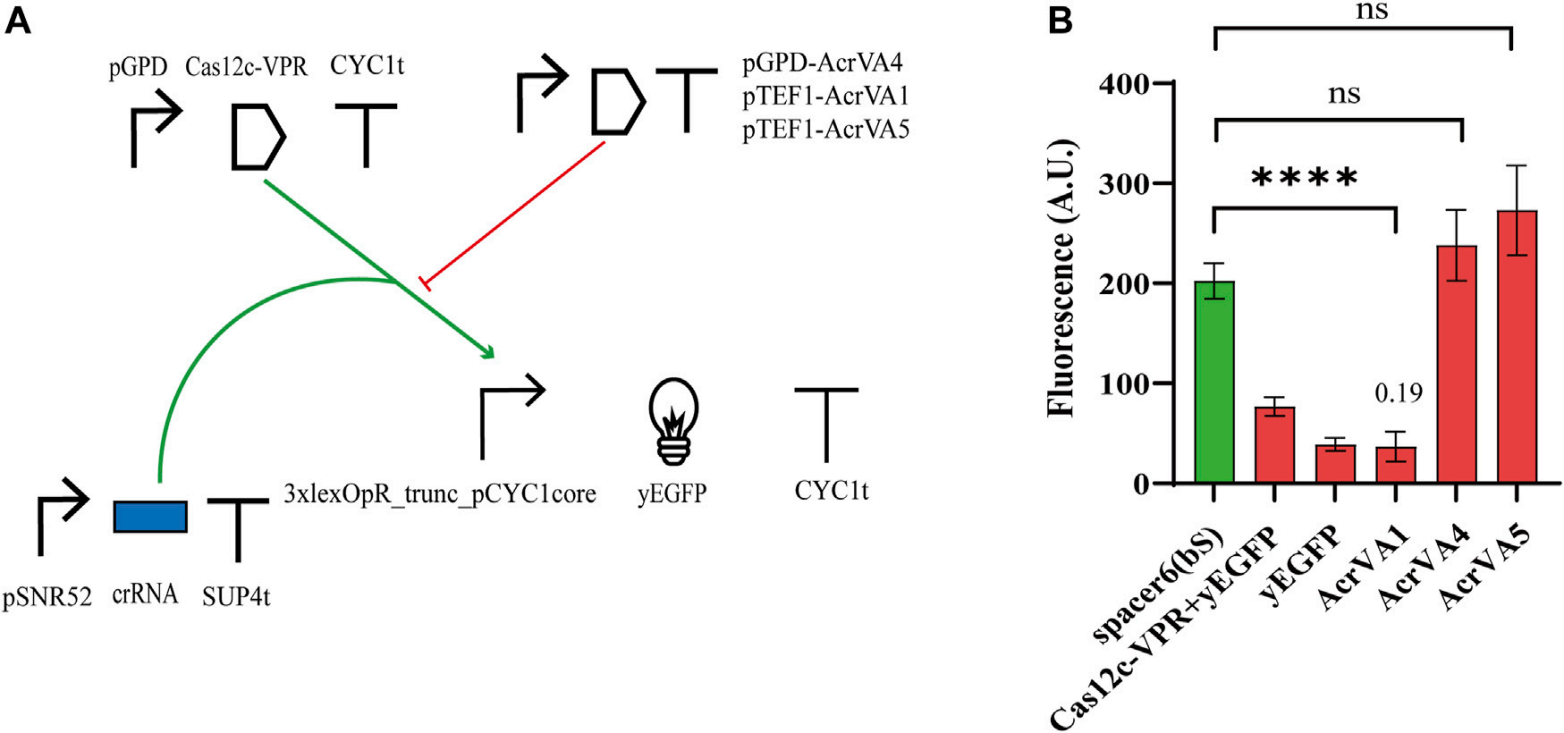
Interaction between Cas12c and three AcrVA proteins. **(A)** Circuit diagram. **(B)** Fluorescence intensity. In the presence of AcrVA1, the fluorescence signal is very close to the basal one from the weak synthetic promoter 3xlexOpR_trunc_pCYC1core. The number on top of the ‘AcrVA1 bar is the OFF/ON ratio corresponds to FI (complete circuit)/FI (control circuit, i.e., without the AcrVA protein) (****, *p*-value <0.0001; ns, *p*-value >0.05; two-sided Welch’s t-test).

The anti-CRISPR (Acr) proteins are a response, evolved by phages, to contrast the CRISPR-Cas immune systems (Tickner et al., 2020). Three type V anti-CRISPR proteins (AcrVA1, AcrVA4, and AcrVA5) have been shown to inhibit strongly the activity of different (d)Cas12a proteins via distinct mechanisms (Yu and Marchisio, 2020). In particular, AcrVA1 prevents the Cas12a:crRNA complex from binding the DNA by mimicking PAM and (sometimes) truncating the crRNA. AcrVA4 hinders the formation of a Cas12a:crRNA:DNA complex by inducing, as a dimer, a “butterfly” configuration made of two Cas12a proteins. AcrVA5, in contrast, works as an acetyltransferase and the acetylated Cas12a is no longer able to interact with PAM. Moreover, AcrVA1 inhibits a broader range of Cas12a homologs compared to AcrVA4 and AcrVA5 (Marino et al., 2018; Yu and Marchisio, 2020). In a previous work from our lab, these three AcrVAs have been shown to inhibit LbCas12a and denAsCas12a (AcrVA1 only) in budding yeast cells (Yu and Marchisio, 2021). Therefore, we checked if they had any effects on Cas12c. We modified Circuit 2 with the addition of a TU expressing an AcrVA protein (see Figure 4A) either under pTEF1 (AcrVA1 and AcrVA5) or pGPD (AcrVA4). The fluorescence intensity measured in the presence of the different AcrVAs (see Figure 4B; Supplementary Table S9) pointed out that AcrVA1 is a potent inhibitor of Cas12c, whereas AcrVA4 and AcrVA5 have no effect whatsoever on it. We speculated that since AcrVA1 mimics PAM (Zhang et al., 2019) and Cas12a PAM (TTTV) contains three possible Cas12c PAMs (TA, TC, and TG), then AcrVA1 should be able to act as a bait also for the system Cas12c:crRNA. It should be noted, though, that FACS experiments first (Supplementary Figure S9), and a viability test later (Supplementary Figure S10; Supplementary Table S10) indicated that AcrVA1 induced mild toxicity in *S. cerevisiae* (viability coefficient: 0.804). This result is analogous to that we reported for the AcrIIA5-SpCas9 interaction (Li et al., 2018).

## Conclusion

In this work, we aimed to ascertain if Cas12c, a type V CRISPR-associated protein that lacks DNase function, could be used as a transcription factor inside *S. cerevisiae* synthetic gene circuits. We started from the construction of simple circuits where Cas12c, either bare or fused to an effector domain, was constitutively expressed to enhance or repress the synthesis of the yEGFP. We found that Cas12c fused to the strong VPR activation domain increased transcription considerably, no matter the DNA strand targeted by its crRNA. The bare Cas12c failed to inhibit yEGFP production. However, when fused to the Mxi1 repression domain, Cas12c-Mxi1 could moderately repress transcription by targeting the sense strand of the promoter (pTEF2) upstream of the *yEGFP* gene. This result showed an opposite behavior to denAsCas12a, the nuclease-deficient type V Cas protein that best serves as a template for engineering transcription factors in yeast. The bare denAsCas12a, indeed, represses fluorescence expression considerably by targeting the middle region of *yEGFP*.

We then tested the working of Cas12c inside hybrid Boolean gates, i.e., logic circuit where both transcription and translation are regulated. In particular, each gate was based on translation inhibition via tetracycline-responsive riboswitches. We built two kinds of hybrid Boolean gates: N-IMPLY, where transcription was activated, and NOR, where transcription was repressed. In general, N-IMPLY gates gave better performance than NOR gates, no matter if the activator was built on Cas12c, denAsCas12a, or LexA-HBD (hER). Moreover, a tandem riboswitch (tc2) usually enhanced the gate performance.

Finally, we checked if Cas12c activity could be controlled by type V anti-CRISPR proteins. We found that AcrVA1 drastically reduced Cas12c (fused to VPR) activity, whereas AcrVA4 and AcrVA5 had no effect on the same chimeric protein. AcrVA1 is a potent inhibitor of denAsCas12a and works as a bait by mimicking the PAM. This can explain its strong impact on Cas12c, whose PAM (TN) is like a piece of that of Cas12a (TTTV). As a drawback, we realized that AcrVA1 induces a mild toxicity in yeast cells (viability coefficient = 0.804). A similar trend was detected also on AcrIIA5 that contrast the activity of dSpCas9.

On the whole, we can claim that Cas12c, fused to an AD, can be used reliably as an activator, even though denAsCas12a appears more performant. Basic engineering of Cas12c as a repressor did not lead to impressive results. Hence, further attempts with different repression domains or longer pre-crRNA (i.e., multiple gene/promoter targeting) should be tried.

By implementing different hybrid gates, we also showed that Cas proteins, when used as templates to construct new transcription factors, can outperform other proteins such as the bacterial repressor LexA. Interestingly, denAsCas12a, i.e., a dead nuclease, regulates transcription better than Cas12c, which naturally silences transcription. A deeper understanding of the Cas12c-based immune system working is probably necessary to explain this apparent contradiction.

## Materials and methods

### Plasmid construction

All plasmids used in this study are listed in Supplementary Table S11. These plasmids were constructed on the pRSII40X yeast integrating shuttle vector collection (Chee and Haase, 2012), where X stands for an auxotrophic marker. We used four of them, namely: HIS3, TRP1, LEU2, and URA3, corresponding to the plasmids: pRSII403/Addgene-35436, pRSII404/Addgene-35438, pRSII405/Addgene-35440, and pRSII406/Addgene-35442, respectively (a gift from Steven Haase).

Two methods were used to construct new plasmids. The first method was enzymatic digestion and ligation. First, the backbone and the insert-containing plasmid were digested overnight. The purified DNA fragments were then ligated using T4 DNA ligase (NEB-M0202S) at 16°C for 8 h. Finally, DNA was eluted from the agarose gel using the AxyPrep DNA Gel Extraction Kit #AP-GX-250. The second method was isothermal assembly. First, standard biological parts, such as promoters, coding regions, and terminators, were extracted and amplified by touchdown PCR using Q5 High-Fidelity DNA Polymerase (NEB-M0491S). The purified PCR products were mixed with a cut-open backbone (i.e., a pRSII40X plasmid stripped of the multiple cloning sequence) in equimolar amount and let in a thermal cycler at 50 C for 1 h (Gibson et al., 2009).

All plasmids constructed in this work were inserted into *E. coli* cells (DH5α, Life Technology 18263-012) and stored in glycerol storage solution. All DNA sequences are reported in Supplementary Table S13. Every plasmid was sequenced by Sanger sequencing at Genewiz Inc., Suzhou, China.

### Yeast transformation

All strains engineered in this work are based on the *S. cerevisiae* strain CEN.PK2-1C (MATa; his3Δ1; leu2-3_112; ura3-52; trp1-289; MAL2-8c; SUC2), Euroscarf (Johann Wolfgang Goethe University, Frankfurt, Germany). For yeast transformation (Daniel Gietz et al., 2002), we followed the PEG/LiAc method. Approximately 5 μg of integrative plasmid were linearized with an appropriate restriction enzyme at the corresponding auxotrophic marker. Transformants were grown on selective synthetic defined medium (2% glucose, 2% agar) at 30°C for 2–3 days. Correct transformants were stored in 15% glycerol storage solutions. All yeast strains realized in this study are listed in Supplementary Table S12.

### Fluorescence measurement

If no inducer was present in the growth solution, yeast cells grew for 16 h at 30°C in SDC supplemented with 2% glucose. If the cells were induced with galactose, the incubation time was of 22 h. If SDC was supplemented with tetracycline, methionine, or copper sulfate, the incubation time was of 22–24 h. Before any FACS experiments, cells were diluted 1:20 (in SDC). To measure fluorescence intensity, we used a BD FACSVerse (blue laser 488 nm, emission filter 527/32). The FACS machine setting was checked via the QC (quality check) procedure by using fluorescent beads (BD FACS quite CS&T Research beads-17495). Each yeast strain was measured three times on different days (independent experiments). In every experiment, for each sample 30000 events were collected at low flow rates (the threshold rate was kept below 2000 events/s). Raw data from the BD FACSVerse were analyzed using the flowcore R-Bioconductor software package (Daniel Gietz et al., 2002).

### Cell viability test with trypan blue

Trypan blue staining assay is a commonly used method to determine cell viability. The assay is based on the principle that trypan blue is excluded from viable cells but penetrates and stains dead cells. In this assay, cells are treated with trypan blue solution, which gives the dead cells a blue color, while viable cells remain unstained. Cells are mixed in a 1:1 ration with 0.4% trypan blue solution. The number of alive and dead cells is then counted under a microscope using a hemocytometer. The percentage of alive cells is calculated by dividing the number of alive cells by the total number of cells.

## Data Availability

The datasets presented in this study can be found in online repositories. The names of the repository/repositories and accession number(s) can be found below: https://flowrepository.org/, http://flowrepository.org/id/RvFrXQ8jGilrL5AUQjr6UWUTFS2qZnklp9JXBP9FTVCoxRSMdOeUK7zVYBGuhOsz.
