# Supplementary material for: Hybrid Boolean gates show that Cas12c controls transcription activation effectively in the yeast S. cerevisiae
Source: Front Bioeng Biotechnol. 2023 Sep 12;11:1267174. doi: 10.3389/fbioe.2023.1267174 (PMC10523329; doi:10.3389/fbioe.2023.1267174)
Supplement: Supplementary file 1 [file DataSheet1.PDF]

SUPPLEMENTARY MATERIAL

Hybrid Boolean gates show that Cas12c controls transcription activation effectively in the yeast S. cerevisiae.

Yifan Liu[1], Huanhuan Ge[1], and Mario Andrea Marchisio[1,*]

[1] School of Pharmaceutical Science and Engineering, Tianjin University, 92 Weijin road, 300072 Tianjin, China

[*] corresponding author. Email addresses: mario@tju.edu.cn or mamarchisio@yahoo.com

Supplementary Figures

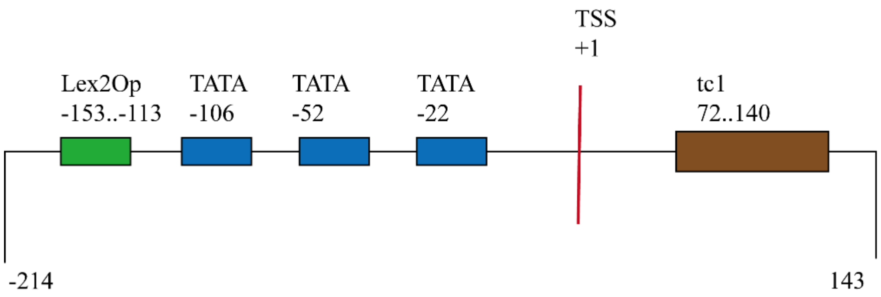

Figure S1. A simple design for a hybrid gate. A single tetracycline-responsive riboswitch (tc1) lies on the 5' UTR, whereas a full lex2Op (to which LexA-based activators bind) is placed upstream of a *CYC1* core promoter.

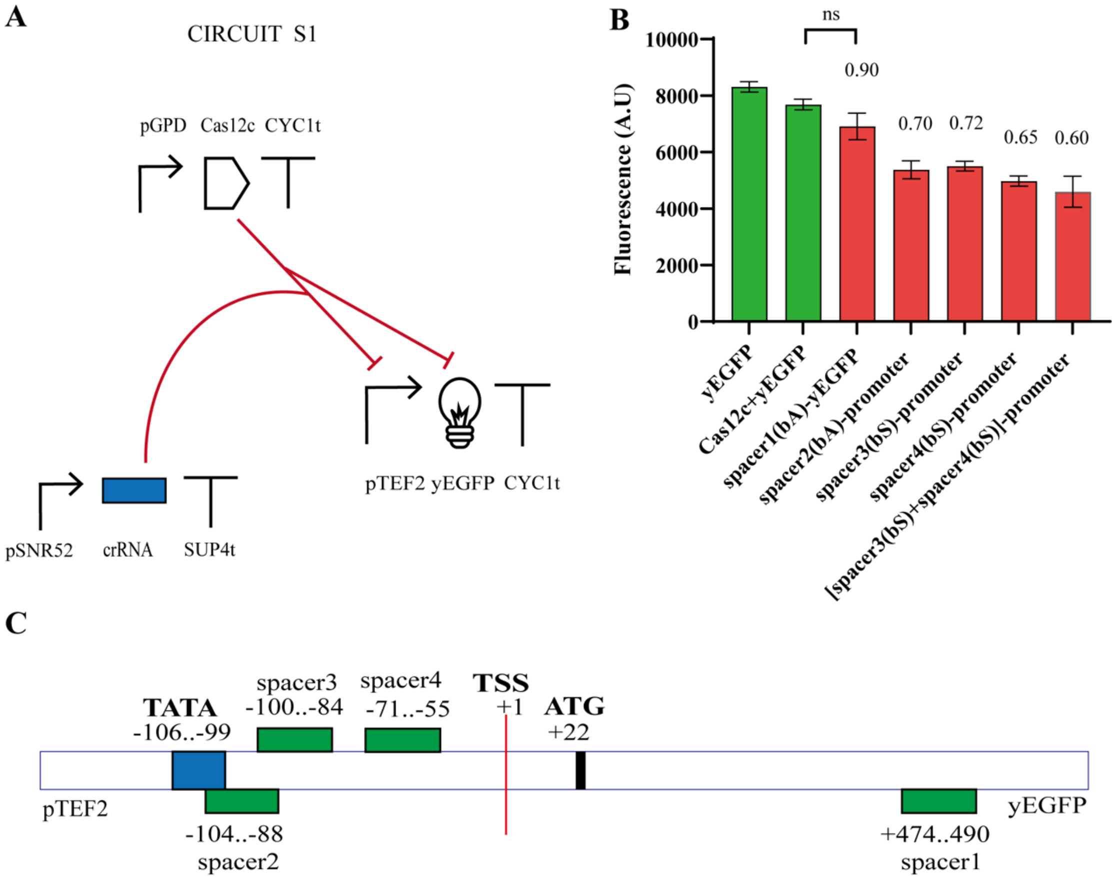

Figure S2. The bare Cas12c does not reduce fluorescence highly in *S. cerevisiae*. (**A**) Circuit S1. Cas12c targets either the *yEGFP* gene or the *TEF2* promoter. The crRNA is expressed via the RNA polymerase III elements pSNR52 and SUP4t. (**B**) Fluorescence intensity. Spacer1 failed to reduce fluorescence in a statistically significant way with respect to the "open" circuit, where the crRNA was not expressed (two-sided Welch's t-test, p-value > 0.05—see Table S1). The numbers on top of the bars are the OFF/ON ratio corresponding to FI (complete circuit)/FI(control circuit, i.e., 'Cas12c+yEGFP'). (**C**) Sites bound by spacer1-4.

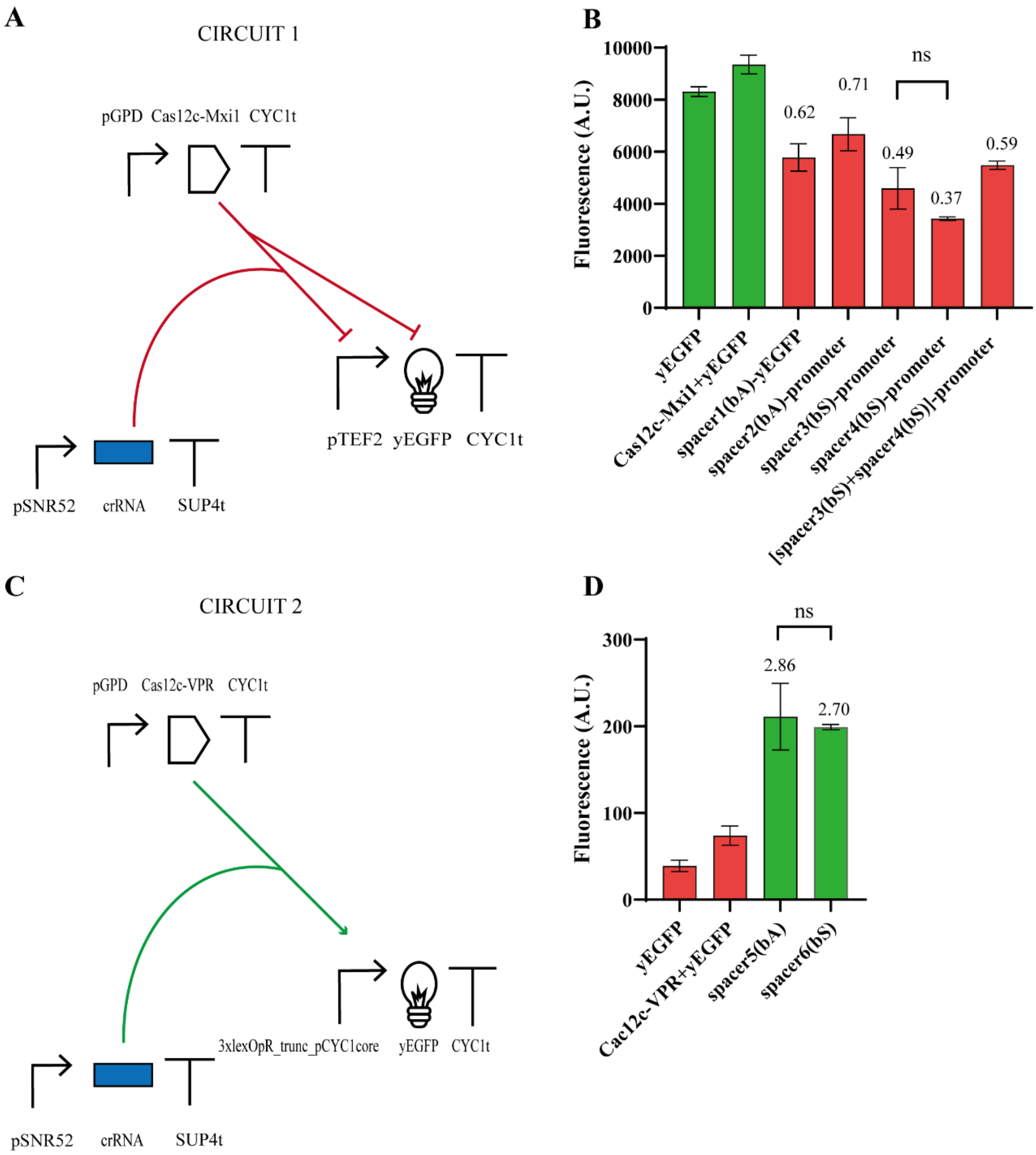

Figure S3. Cas12c becomes a transcription factor upon fusion to an effector domain. (**A**) Circuit 1 diagram. Cas12c-Mxi1 targets either pTEF2 or the sequence of the *yEGFP* gene. The hammer-like red line indicates repression of transcription. (**B**) Fluorescence intensity (FI). The numbers on top of the bars are the OFF/ON ratio that corresponds to FI (complete circuit)/FI (control circuit, i.e., without the crRNA expression cassette). (**C**) Circuit 2 diagram. Green fluorescence expression is controlled by the synthetic promoters 3xlexOpR_trunc_pCYC1core. A green arrow stands for activation of transcription. (**D**) Fluorescence intensity. The numbers on top of the bars are the ON/OFF ratio corresponding to FI (complete circuit)/FI (control circuit). ‘ns’: no statistically significant difference (p-value > 0.05; two-sided Welch’s t-test).

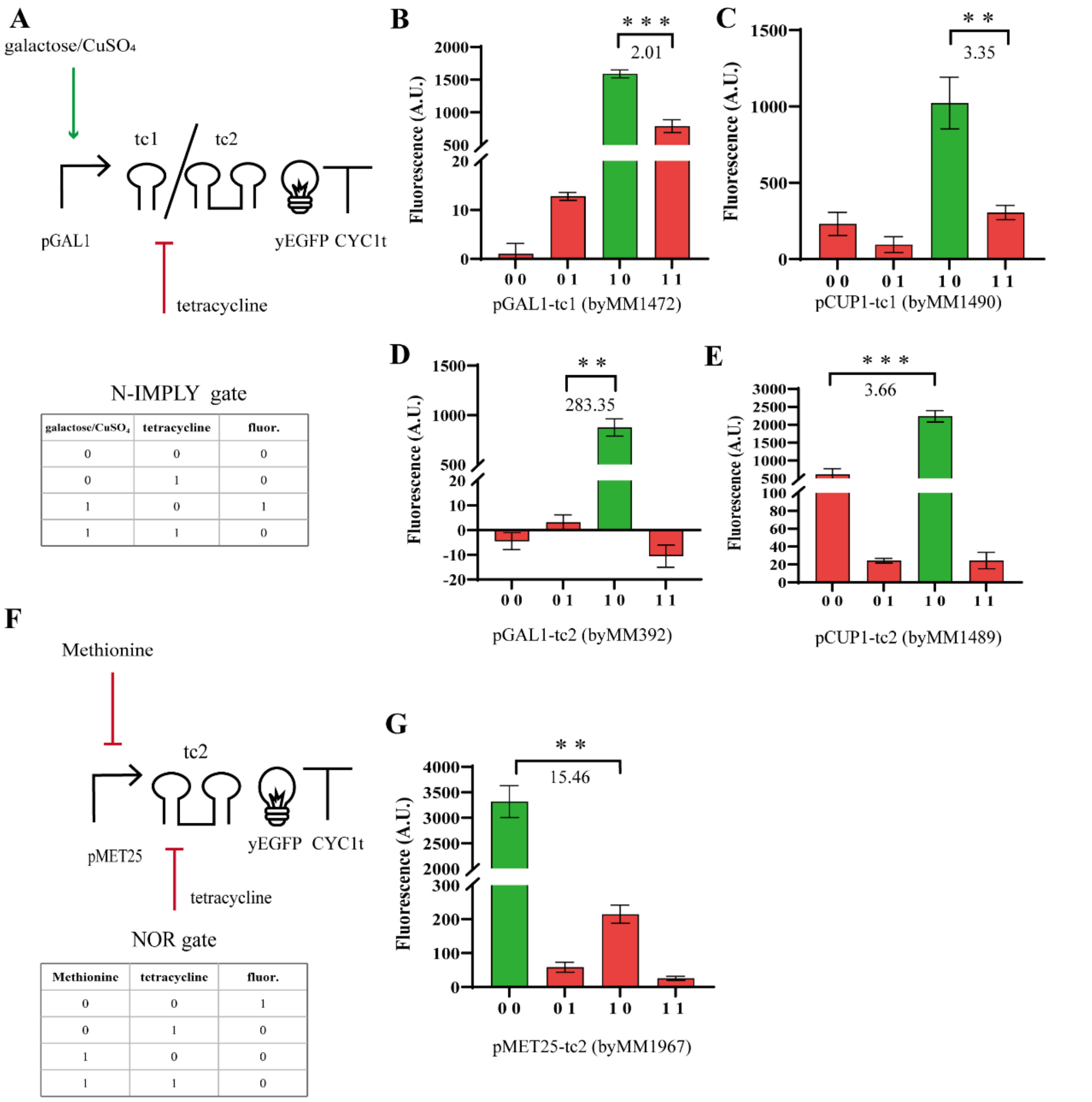

galactose/CuSO4	tetracycline	fluor.
0	0	0
0	1	0
1	0	1
1	1	0

Methionine	tetracycline	fluor.
0	0	1
0	1	0
1	0	0
1	1	0

Figure S4. Two-input hybrid Boolean gates built on a single transcription unit. (**A**) Scheme of the N-IMPLY gates (galactose AND NOT tetracycline); ($CuSO_4$ AND NOT tetracycline). (**B, C, D, E**) Fluorescence intensity from the combination of pGAL1 with tc1; pCUP1 with tc1; pGAL1 with tc2; and pCUP1 with tc2, respectively. (**F**) NOR gate scheme (NOT (methionine OR tetracycline)). (**G)** NOR gate fluorescence intensities (pMET25 and tc2). Notice that the '1' concentrations of the different input signals were: 130 μM tetracycline, 10 mM methionine[42], 1 mM $CuSO_4$, and 2% galactose in SDC (synthetic defined complete) medium. The numbers on the bar charts are the ρ values (**, p-value < 0.01; ***, p-value < 0.001; two-sided Welch's t-test).

A

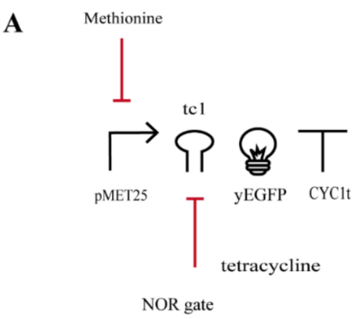

Methionine	tetracycline	fluor.
0	0	1
0	1	0
1	0	0
1	1	0

B

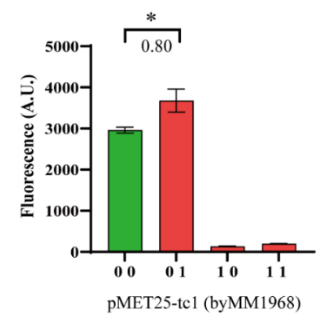

Figure S5. The NOR gate (NOT (methionine OR tetracycline)) with tc1 failed to reproduce its truth table. Tetracycline appeared not effective on a single riboswitch. The number on the bar chart is the ρ value (*, p-value < 0.05; t-sided Welch's t-test—see Table S4).

A

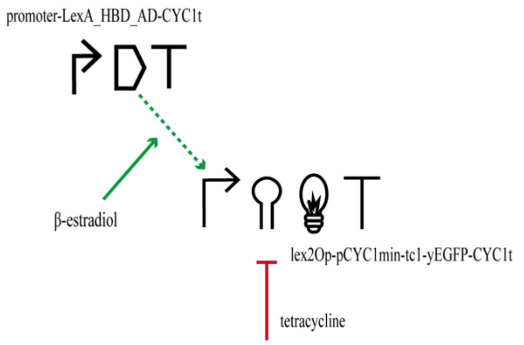

estradiol	tetracycline	fluor.
0	0	0
0	1	0
1	0	1
1	1	0

B

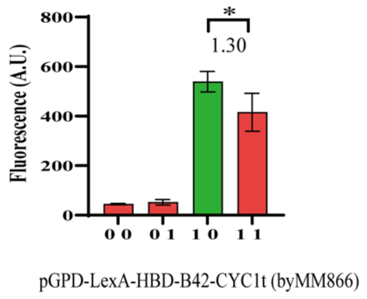

C

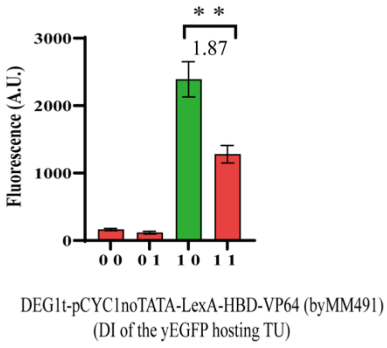

Figure S6. N-IMPLY gates sensing tetracycline and β-estradiol. (**A**) Circuit schemes. The TU expressing yEGFP always contains a single lex2Op and tc1. In contrast, the TU hosting the chimeric activator could use either DEG1t-pCYC1noTATA together with VP64 or pGPD and B42. (**B**) The design with pGPD and B42 returns a very low ρ-value: 1.3. (**C**) A double integration of the TU expressing yEGFP does not improve the ρ-value of the circuit in Figure 4C-D. The numbers on the bar charts are the ρ values (*, p-value < 0.05; **, p-value < 0.01; two-sided Welch's t-test—see Table S5).

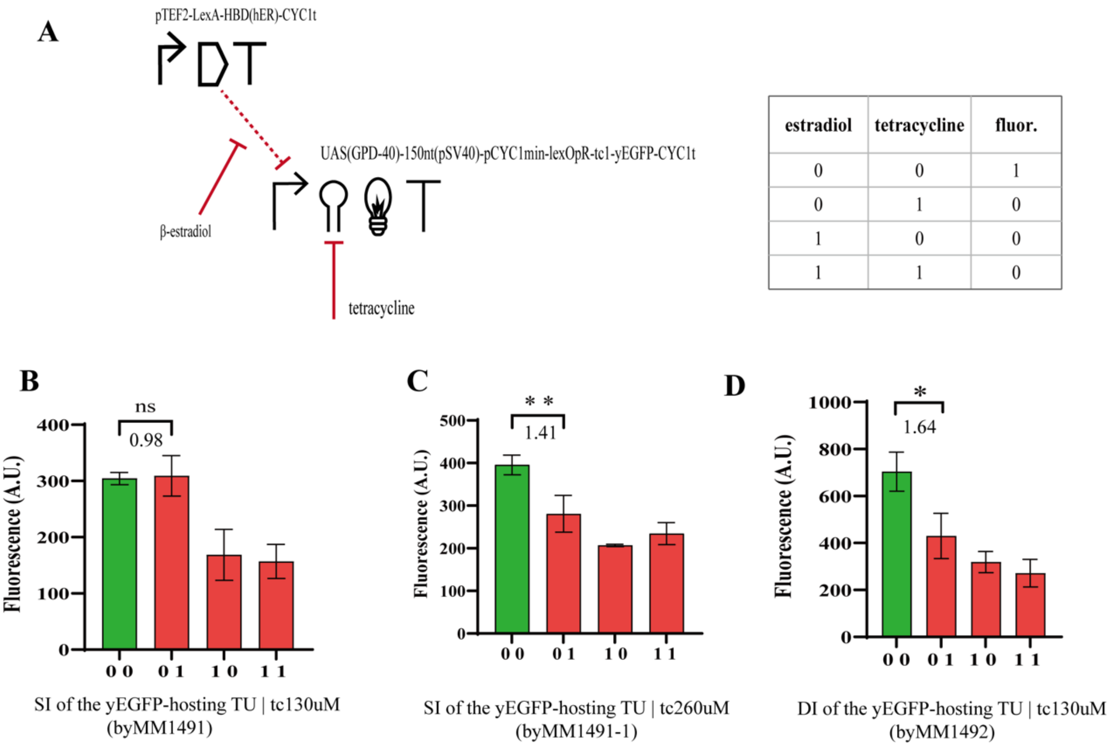

estradiol	tetracycline	fluor.
0	0	1
0	1	0
1	0	0
1	1	0

Figure S7. NOR gates sensing tetracycline (via tc1) and β-estradiol. (**A**) Circuit diagram. (**B, C**) A single copy of the TU expressing yEGFP demands 260 µM tetracycline to show a significant statistical difference between the only 1 output and MAX(0). (**D**) With a double integration of the same TU, 130µM tetracycline are enough to have p-value < 0.05. However, the ρ-value is equal to 1.64 only (*, p-value < 0.05; **, p-value < 0.01; ns, p-value > 0.05; two-sided Welch's t-test—see Table S6).

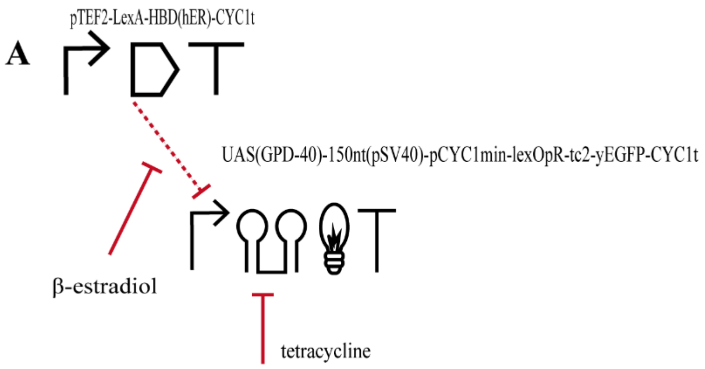

estradiol	tetracycline	fluor.
0	0	1
0	1	0
1	0	0
1	1	0

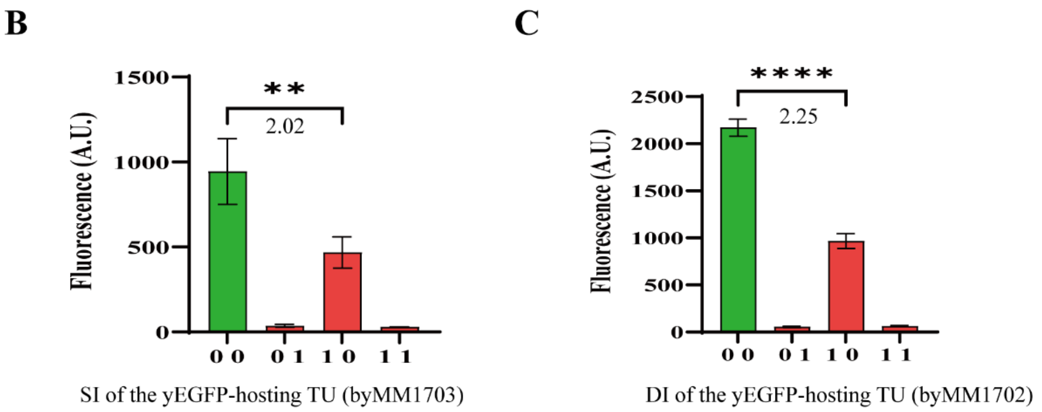

Figure S8. NOR gates responding to tetracycline and β-estradiol. (**A**) Circuit scheme. LexA-HBD(hER) is constitutively expressed by the rather strong *TEF2* promoter. The green fluorescent signal is controlled by the synthetic promoter UAS(GPD-40)-150nt(pSV40)-pCYC1min-lexOpR that carries tc2 on the 5' UTR. (**B, C**) Fluorescence intensity. byMM1702 contains a double integration of the yEGFP-hosting TU, whereas byMM1703 has just a single copy of it. The input concentrations are: 1000 nM β-estradiol, and 130 μM tetracycline. The numbers on the bar charts are the ρ values (**, p-value < 0.01; ****, p-value < 0.0001; two-sided Welch's t-test).

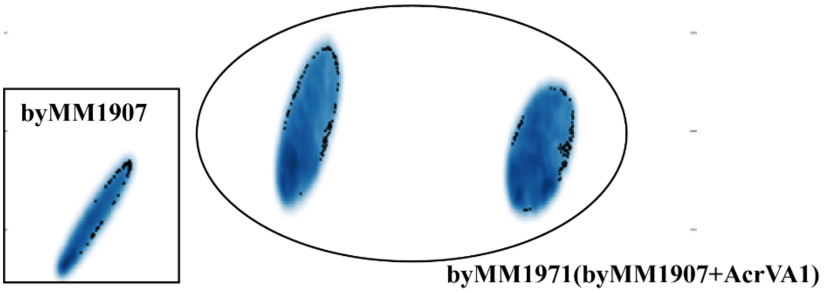

Figure S9. The shape of cell populations without (byMM1907) and with (byMM1971) AcrVA1. The latter appear irregular, which is a hint of mild toxicity in the cells.

square1

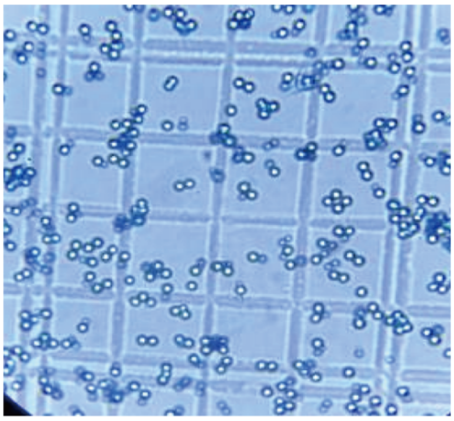

square2

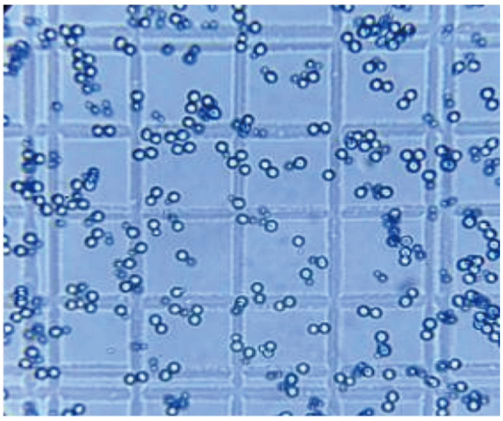

square3

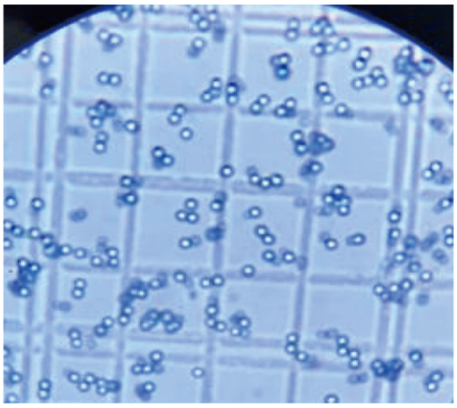

square4

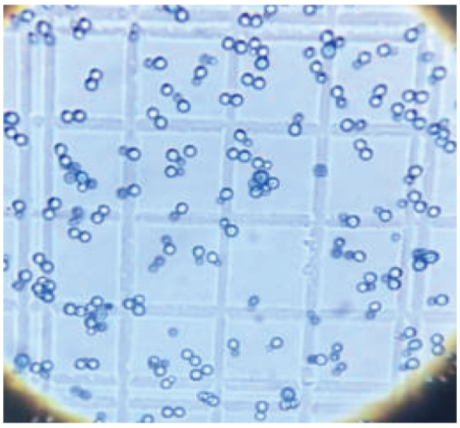

square5

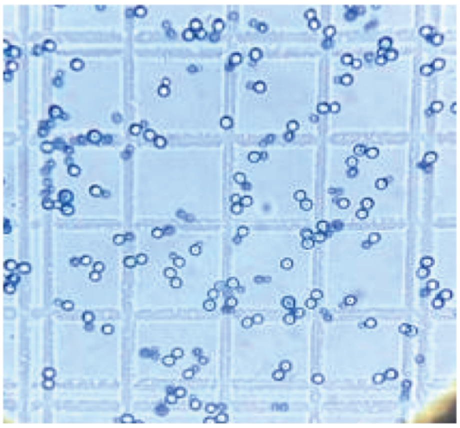

Figure S10. Trypan Blue viability test. Images are taken from a AOSVI microscope, 40x magnification.

Supplementary Tables

Table SP. Mean fluorescence, in arbitrary units (A.U.), expressed by constitutive natural and synthetic yeast promoters. SD: standard deviation. 'Ratio vs pGPD' is calculated by dividing the fluorescence expressed by each promoter by that of pGPD. Both mean fluorescence and SD are calculated on three independent measurements.

Promoter	Average fluorescence (A.U.)	SD	Ratio vs pGPD
pGPD	18390.48	1210.51	1.00
pTEF2	8063.26	56.68	0.44
DEG1t-pCYC1noTATA	3631.45	175.24	0.20

Table S1. Circuit S1 mean fluorescence intensity (FI). SD is the standard deviation of the mean. Replicates are the number of independent experiments that were carried out. The p-value was calculated via two-sided Welch's t-test to compare the fluorescence level of the full circuit with that of the control circuit lacking the crRNA expression cassette. The OFF/ON ratio corresponds to FI(complete circuit)/FI(control circuit).

Strains	Content	Mean fluorescence intensity	SD	Replicates	p-value	p-value summary	OFF/ON ratio
byMM1202	yEGFP	8308.45	186.27	6			
byMM1708	Cas12c +yEGFP	7683.41	188.37	7			
byMM1845	spacer1(bA)-yEGFP	6908.50	469.78	3	0.0950	ns	0.90
byMM1714	spacer2(bA)-promoter	5373.82	322.14	3	0.0026	**	0.70
byMM1717	spacer3(bS)-promoter	5501.02	170.53	3	<0.0001	****	0.72
byMM1842	spacer4(bS)-promoter	4973.98	180.36	3	<0.0001	****	0.65
byMM1860	[spacer3(bS)+spacer4(bS) -promoter	4595.32	547.49	3	0.0078	**	0.60

The four circuits where fluorescence was significantly repressed (spacer2(bA)-promoter, spacer3(bS)-promoter, spacer4(bS)-promoter, spacer3(bS)+spacer4(bS)-promoter) are not statistically equivalent: p-value: 0.0428 (*), one-way ANOVA.

Table S2. Circuit 1 mean fluorescence intensity (FI). SD is the standard deviation of the mean. Replicates are the number of independent experiments. The p-value was calculated via two-sided Welch's t-test to compare the fluorescence intensity of the full circuit with that of the control circuit lacking the crRNA expression cassette. The OFF/ON ratio corresponds to FI(complete circuit)/FI(control circuit).

Strains	Content	Mean fluorescence intensity	SD	Replicates	p-value	p-value summary	OFF/ ON ratio
byMM1202	yEGFP	8308.45	186.27	6			
byMM1867	Cas12-Mxi1+yEGFP	9351.75	359.07	3			
byMM1885	spacer1(bA)- yEGFP	5776.73	524.23	3	0.0011	**	0.62
byMM1880	spacer2(bA)-prompter	6672.64	634	3	0.0067	**	0.71
byMM1886	spacer3(bS)-promoter	4596.62	797	3	0.0034	**	0.49
byMM1887	spacer4(bS)-promoter	3430.18	67	3	0.0009	***	0.37
byMM1882	[spacer3(bS)+spacer4(bS) -promoter	5482.54	163	3	0.0007	***	0.59

Comparison between spacer3(bS)-promoter and spacer4(bS)-promoter: p-value: 0.1257 (ns), two-sided Welch's t-test.

Table S3. Circuit 2 mean fluorescence intensity (FI). SD is the standard deviation of the mean. Replicates refer to the number of independent experiments. The p-value was calculated via two-sided Welch's t-test to compare the fluorescence intensity of the full circuit with that of the control circuit lacking the crRNA expression cassette. The ON/OFF ratio corresponds to FI(complete circuit)/FI(control circuit).

Strains	Content	Mean fluorescence intensity	SD	Replicates	p-value	p-value summary	ON/ OFF ratio
byMM556	yEGFP	38.88	6.51	3			
byMM1896	Cas12c-VPR +yEGFP	73.82	11.04	3			
byMM1903	spacer5(bA)	211.02	38.55	3	0.0189	*	2.86
byMM1907	spacer6(bS)	198.95	2.93	3	0.0015	**	2.70

Comparison between spacer6(bS) and spacer5(bA): p-value: 0.6423 (ns), two-sided Welch's t-test.

Table S4. Analysis of the N-IMPLY and NOR gates made of a single TU. Mean FI: mean fluorescence intensity. SD: standard deviation of the mean. Replicates: number of independent measurements (i.e., done on different days). ρ-value is the ratio between the only 1 and MAX(0) output. p-value is calculated, via a two-sided Welch's t-test, between the only 1 and MAX(0) output.

Strains	Content		N-imply gate (galactose AND NOT tetracycline)				p-value	p-value summary	ρ-value
			0	0	1	0			
			0 0	0 1	1 0	1 1			
byMM1472	pGAL1-tc1-yEGFP	Mean-FI	1.04	12.76	1589.25	789.09	0.0008	***	2.01
		SD	2.10	0.81	59.83	99.02			
		Replicates	3	3	3	3			
byMM392	pGAL-tc2-yEGFP	Mean-FI	-4.50	3.09	874.45	-10.56	0.0033	**	283.35
		SD	3.40	3.06	87.45	4.49			
		Replicates	3	3	3	3			

Strains	Content		N-imply gate ($CuSO_4$ AND NOT tetracycline)				p-value	p-value summary	ρ-value
			0	0	1	0			
			0 0	0 1	1 0	1 1			
byMM1490	pCUP1-tc1-yEGFP	Mean-FI	230.3	94.9	1021.95	304.94	0.0022	**	3.35
		SD	76.07	52.25	169.3	47.32			
		Replicates	3	3	4	3			
byMM1489	pCUP1-tc2-yEGFP	Mean-FI	610.17	24.36	2234.66	24.5	0.0001	***	3.66
		SD	163.43	2.65	157.24	9.13			
		Replicates	3	3	4	4			

Strains	Content		NOR gate (NOT Methionine OR tetracycline)				p-value	p-value summary	ρ-value
			1	0	0	0			
			0 0	0 1	1 0	11			
byMM1968	pMET25-tc1-yEGFP	Mean-FI	2958.88	3678	133.62	197.09	0.0404	*	0.80
		SD	70.37	281	10.21	8.31			
		Replicates	3	3	4	3			
byMM1967	pMET25-tc2-yEGFP	Mean-FI	3316.85	58.25	214.55	25.71	0.0032	**	15.46
		SD	313.76	14.73	26.89	6.07			
		Replicates	3	3	3	3			

Table S5. Analysis of the N-IMPLY gates responding to tetracycline and β-estradiol. Mean-FI: mean fluorescence intensity. SD: standard deviation of the mean. Replicates: the number of independent measurements. ρ-value: the ratio between the only 1 and the MAX(0) output. The p-value is calculated via two-sided Welch's t-test to compare the only 1 and MAX(0) output.. P1 represents the promoter "lex2Op-pCYC1min". P2 is the promoter "3xlex2Op-pCYCImin". SI means single integration. DI stands for double integration.

Strains	Content		N-imply gate (estradiol AND NOT tetracycline)				p-value	p-value summary	ρ-value
			0	0	1	0			
			0 0	0 1	1 0	1 1			
byMM866	pGPD-LexA-HBD-B42 +P1-tc1	Mean -FI	44.87	52.17	538.83	415.55	0.0402	*	1.3
		SD	2.92	11.05	41.3	76.68			
		Replicates	4	4	4	4			
byMM492	DEG1t-pCYC1noTATA-LexA-HBD-VP64 +P1-tc1(SI)	Mean-FI	81.81	74.38	1306.62	542.48	0.0067	**	2.41
		SD	9.98	13.25	170.49	277.61			
		Replicates	4	4	3	4			
byMM491	DEG1t-pCYC1noTATA-LexA-HBD-VP64 +P1-tc1(DI)	Mean -FI	162.88	116.78	2389.78	1278.07	0.0010	**	1.87
		SD	15.51	19.92	260.29	129.84			
		Replicates	3	4	4	3			
byMM1479	DEG1t-pCYC1noTATA-LexA-HBD-VP64 +P2-tc2	Mean -FI	68.55	9.86	1135.75	22.28	0.0002	***	16.57
		SD	13.42	4.81	94.89	10.22			
		Replicates	4	4	4	4			

Table S6. Analysis of the NOR gates responding to tetracycline and β-estradiol. Mean-FI: mean fluorescence intensity. SD: the standard deviation of the mean. Replicates: the number of independent experiments. ρ-value: the ratio between the only 1 and MAX(0) output. The p-value is calculated via two-sided Welch's t-test to compare the only 1 and MAX(0) output. P3 represents the promoter "UAS(GPD-40)-150nt (pSV40)-pCYC1min-lexOpR". SI means single integration. DI, double integration.

Strains	Content		NOR gate (NOT (estradiol OR tetracycline))				p-value	p-value summary	ρ-value
			1	0	0	0			
			0 0	0 1	1 0	1 1			
byMM1491	pTEF2-LexA-HBD +P3-tc1(SI)	Mean-FI	304.34	309.08	168.49	156.80	0.8450	ns	0.98
		SD	10.72	36.07	45.34	30.22			
		Replicates	3	3	3	3			
byMM1492	pTEF2-LexA-HBD +P3-tc1t(DI)	Mean-FI	703.83	429.91	318.70	271.53	0.0212	*	1.64
		SD	83.04	96.55	45.23	58.72			
		Replicates	3	3	3	3			
byMM1491-1	pTEF2-LexA-HBD +P3-tc1(SI)	Mean-FI	395.45	280.72	206.72	234.36	0.0066	**	1.41
		SD	23.04	43.04	2.84	25.82			
		Replicates	4	4	3	3			
byMM1703	pTEF2-LexA-HBD +P3-ext_tc2(SI)	Mean-FI	944.54	35.50	467.48	29.28	0.0095	**	2.02
		SD	193.49	9.02	92.45	1.11			
		Replicates	4	3	4	3			
byMM1702	pTEF2-LexA-HBD +P3-ext_tc2(DI)	Mean-FI	2169.43	54.34	965.85	62.35	<0.0001	****	2.25
		SD	92.05	6.91	78.10	6.42			
		Replicates	4	5	4	3			

Table S7. Analysis of the N-IMPLY gates—based on type V CRISPR-Cas systems—responding to galactose and tetracycline. Mean-FI: mean fluorescence intensity. SD: the standard deviation of the mean. Replicates: the number of independent measurements. ρ-value: the ratio between the only 1 and MAX(0) output. The p-value is calculated via two-sided Welch's t-test to compare the only 1 and MAX(0) output.

Strains	Content		N-IMPLY gate (galactose AND NOT tetracycline)				p-value	p-value summary	ρ-value
			0	0	1	0			
			0 0	0 1	1 0	1 1			
byMM1899	ext_tc2-GFP +pGAL1-denAsCas12-yoVPR +spacer7(bA)	Mean-FI	206.74	35	1305.48	139.03	0.0030	**	6.31
		SD	23.62	1.4	119.69	9.78			
		Replicates	3	3	3	3			
byMM1916	ext_tc2-GFP+pGAL1-cas12c-yoVPR+spacer5(bA)	Mean-FI	386.88	60.6	1558.28	167.6	0.0012	**	4.03
		SD	75.63	18.22	9.67	16.17			
		Replicates	3	3	4	3			

Table S8. Analysis of the NOR gates—based on type V CRISPR-Cas systems—responding to galactose/crRNA and tetracycline. Mean-FI: mean fluorescence intensity. SD: the standard deviation of the mean. Replicates: the number of independent measurements. ρ-value: the ratio between the only 1 and MAX(0) output. The p-value is calculated via two-sided Welch's t-test to compare the only 1 and MAX(0) output.

Strains	Content		NOR gate (NOT (galactose/crRNA OR tetracycline))				p-value	p-value summary	ρ-value
			1	0	0	0			
			0 0	0 1	1 0	1 1			
byMM1888	pGAL1-denAsCas12a +ext_tc2-GFP+spacer8(bA)	Mean-FI	3195.76	87.31	1033.75	-10.69	0.0021	**	3.09
		SD	318.07	8.62	152.92	8.2			
		Replicates	3	3	3	3			
byMM1969	pGPD-cas12c-Mxi1 +ext_tc2GFP+spacer4(bS)	Mean-FI	2865.56	96.64	1693.64	97.14	0.0115	*	1.69
		SD	295.17	20.8	133.06	5.39			
		Replicates	3	3	4	4			

Table S9. Analysis of the circuits expressing AcrVA proteins—mean fluorescence intensity (FI) measurements. SD: standard deviation of the mean. Replicates: the number of independent experiments. The p-value was calculated via two-sided Welch's t-test to compare the fluorescence intensity of the full circuit with that lacking the AcrVA expression cassette (control circuit). The OFF/ON ratio corresponds to FI(complete circuit)/FI(control circuit—without AcrVA protein).

Strains	Content	Mean fluorescence intensity	SD	Replicates	p-value	p-value summary	OFF/ON Ratio
byMM556	yEGFP	38.88	6.51	3			
byMM1896	Cas12c-VPR +yEGFP	73.82	11.04	3			
byMM1907	Spacer6(bS)	198.95	2.93	3			
byMM1971	AcrVA1	36.95	14.98	3	<0.0001	****	0.19
byMM1965	AcrVA4	238.00	35.62	3	0.2180	ns	1.20
byMM1970	AcrVA5	273.03	44.72	3	0.1018	ns	1.37

Table S10. Cell viability test.

	Square1	Square2	Square3	Square4	Square5	average	sd
alive	72	63	54	53	66	61.6	7.23
dead	18	16	14	17	10	15	2.83
Cell viability	0.8	0.8	0.79	0.76	0.87	0.804	0.036

Cell viability = 61.6/(61.6+15) = 80.4%

Table S11. All integrative plasmids used in this work.

Plasmid name	Construct
pMM577	pRSII406-pGAL1-tc2-GFP-CYC1
pMM1113	pRSII406-pGAL1-tc1-yEGFP-CYC1t
pMM1457	pRSII406-pCUP1-tc2-GFP-CYC1t
pMM1458	pRSII406-pCUP1-tc1-yEGFP-CYC1t
pMM1602	pRSII405-pmet25-tc2-GFP-CYC1t
pMM1601	pRSII405-pmet25-tc1-yEGFP-CYC1t
pMM665	pRSII406-lex2Op-pCYC1min-tc1-yEGFP-CYC1t
pMM363	pRSII405-DEG1t-pCYC1noTATA-LexA-HBD-VP64-CYC1t
pMM403	pRSII405-pGPD-LexA-HBD-B42
pMM619	pRSII406-3lex2Op-pCYC1min-tc2-GFP-CYCIt
pMM1178	pRSII406-UAS(GPD-40)-150nt(pSV40)-pCYC1min-lexOpR-tc1-yEGFP-CYCIt
pMM643	pRSII405-pTEF2-LexA-HBD-STOP-CYC1t
PMM1500	pRSII406-UAS(GPD-40)-150nt(pSV40) pCYC1min-lexOpR-ext_tc2-GFP-CYC1t

pMM1123	pRSII405-pTEF2-yEGFP-HA_tag-CYC1t
pMM1531	pRSII406-pGPD-ATG-NLS-GS-HIStag-GS-BamHI-Cas12c-XhoI-GS-NLS-TAA-CYC1t
pMM1533	PRSII404-PSNR52-DR(cas12c)-spacer1-SUP4t
pMM1550	PRSII404-PSNR52-DR(cas12c)-spacer2-SUP4t
pMM1561	PRSII404-PSNR52-DR(cas12c)-spacer3-SUP4t
pMM1563	PRSII404-PSNR52-DR(cas12c)-spacer4-SUP4
pMM1579	PRSII404-PSNR52-DR(cas12c)-spacer3+spacer4-SUP4t
pMM1369	pMM47-pGPD-ATG-NLS-GS-HIStag-GS-BamHI-dCasX-XhoI-linker_GS-NLS_Mxi1_GS-TAA-CYC1t
pMM1572	pMM47-pGPD-ATG-NLS-GS-HIStag-GS-BamHI-cas12c-XhoI-linker_GS-NLS_Mxi1_GS-TAA-CYC1t
pMM815	pRSII406-pGPD-ATG-NLS-GS-HIStag-GS-BamHI-denAsCas12a-XhoI-linker_yoVPR_ NLS-TAA-mTGuo1
pMM729	pRSII405-3xlexOpR_trunc_pCYC1core-yEGFP-Tsynth24
pMM1571	pRSII406-pGPD-ATG-NLS-GS-HIStag-GS-BamHI-cas12c-XhoI-linker_yoVPR_NLS-TAA-mTGuo1
pMM1570	PRSII404-PSNR52-DR(cas12c)-spacer5-SUP4t
pMM1573	PRSII404-PSNR52-DR(cas12c)-spacer6-SUP4t
pMM776	pRSII406-pGAL1-ATG-NLS-GS-HIStag-GS-BamHI-denAsCas12a-XhoI-linker _yoVPR_ NLS-TAA-mTGuo1
pMM779	pRSII404-pSNR52-DR(AsCas12a)_spacer7-SUP4t
pMM1535	pRSII405- 5xlexOpR-pCYCImin-tc2-GFP-CYCIt
pMM1599	pRSII406-pGAL1-ATG-NLS-GS-HIStag-GS-BamHI-Cas12c-XhoI-linker_yoVPR_NLS-TAA-mTGuo1
pMM878	pMM47-pGAL1-ATG-NLS-GS-HIStag-GS-BamHI-denAsCas12a-XhoI-GS-NLS-TAA-CYC1t
pMM938	pRSII405-pSNR52-DR(AsCas12a)-spacer8-SUP4t
pMM1578	pRSII404-pTEF2-ext_tc2-GFP-CYC1t
pMM1603	PRSII405-PSNR52-DR(cas12c)-spacer4-SUP4t
pMM937	pRSII403-pGPD-ATG_NLS_GS_FLAGtag_GS-BamHI-yo_AcrVA4-XhoI_GS_NLS_ TAA-CYC1t
pMM987	pRSII403-pTEF1-ATG_NLS_GS_FLAGtag_GS-BamHI-yo_AcrVA5-XhoI_GS_NLS_ TAA-CYC1t
pMM1085	pRSII403-pTEF1-ATG_NLS_GS_FLAGtag_GS-BamHI-yo_AcrVA1-XhoI_GS_NLS_ TAA-Tsynth6

Table S12. All synthetic yeast strains realized in this work.

Strain name	Strain genotype
byMM392	byMM111 pMM577::URA3
byMM1472	byMM584 pMM1113::URA3
byMM1489	byMM584 pMM1457::URA3
byMM1490	byMM584 pMM1458::URA3
byMM1967	byMM584 pMM1602::LEU2
byMM1968	byMM584 pMM1601::LEU2
byMM866	byMM584 pMM665::URA3 pMM403::LEU2
byMM491	byMM111 pMM363::LEU2 pMM665::URA3
byMM492	byMM111 pMM363::LEU2 pMM665::URA3
byMM1479	byMM584 pMM619::URA3 pMM363::LEU2
byMM861	byMM584 pMM665::URA3
byMM451	byMM111 pMM363::LEU2
byMM932	byMM584 pMM619::URA3
byMM1486	byMM584 pMM1178::URA3
byMM1487	byMM584 pMM1178::URA3
byMM1491	byMM584 pMM1178::URA3 pMM643::LEU2
byMM1492	byMM584 pMM1178::URA3 pMM643::LEU2
byMM1701	byMM584 pMM1500::URA3
byMM1700	byMM584 pMM1500::URA3
byMM1702	byMM584 pMM1500::URA3 pMM643::LEU2
byMM1703	byMM584 pMM1500::URA3 pMM643::LEU2
byMM1202	byMM584 pMM1123::LEU2
byMM1708	byMM584 pMM1123::LEU2 pMM1531::URA3
byMM1845	byMM584 pMM1123::LEU2 pMM1531::URA3 pMM1533::TRP1
byMM1714	byMM584 pMM1123::LEU2 pMM1531::URA3 pMM1550::TRP1
byMM1717	byMM584 pMM1123::LEU2 pMM1531::URA3 pMM1561::TRP1
byMM1842	byMM584 pMM1123::LEU2 pMM1531::URA3 pMM1563::TRP1
byMM1860	byMM584 pMM1123::LEU2 pMM1531::URA3 pMM1579::TRP1
byMM1867	byMM584 pMM1123::LEU2 pMM1572::URA3
byMM1885	byMM584 pMM1123::LEU2 pMM1572::URA3 pMM1533::TRP1
byMM1880	byMM584 pMM1123::LEU2 pMM1572::URA3 pMM1550::TRP1
byMM1886	byMM584 pMM1123::LEU2 pMM1572::URA3 pMM1561::TRP1
byMM1887	byMM584 pMM1123::LEU2 pMM1572::URA3 pMM1561::TRP1
byMM1882	byMM584 pMM1123::LEU2 pMM1572::URA3 pMM1579::TRP1
byMM556	byMM584 pMM729::LEU2
byMM1896	byMM584 pMM729::LEU2 pMM1571::URA3
byMM1903	byMM584 pMM729::LEU2 pMM1571::URA3 pMM1570::TRP1
byMM1907	byMM584 pMM729::LEU2 pMM1571::URA3 pMM1573::TRP1
byMM1908	byMM584 pMM729::LEU2 pMM1571::URA3 pMM1573::TRP1
byMM1861	byMM584 pMM1535::LEU2
byMM1898	byMM584 pMM1535::LEU2 pMM776::URA3
byMM1899	byMM584 pMM1535::LEU2 pMM776::URA3 pMM779::TRP1

byMM1910	byMM584 pMM1535::LEU2 pMM1599::URA3
byMM1915	byMM584 pMM1535::LEU2 pMM1599::URA3 pMM1570::TRP1
byMM1916	byMM584 pMM1535::LEU2 pMM1599::URA3 pMM1570::TRP1
byMM1878	byMM584 pMM1578::TRP1
byMM1883	byMM584 pMM1578::TRP1 pMM878::URA3
byMM1888	byMM584 pMM1578::TRP1 pMM878::URA3 pMM938::LEU2
byMM1889	byMM584 pMM1578::TRP1 pMM878::URA3 pMM938::LEU2
byMM1909	byMM584 pMM1578::TRP1 pMM1572::URA3
byMM1969	byMM584 pMM1578::TRP1 pMM1572::URA3 pMM1603::LEU2
byMM1965	byMM584 pMM729::LEU2 pMM1571::URA3 pMM1573::TRP1 pMM937::HIS3
byMM1970	byMM584 pMM729::LEU2 pMM1571::URA3 pMM1573::TRP1 pMM987::HIS3
byMM1971	byMM584 pMM729::LEU2 pMM1571::URA3 pMM1573::TRP1 pMM1085::HIS3

Table S13. DNA sequences used in this work.

DNA fragments	sequence
pGAL1	ATATACATATCCATATCTAATCTTACTTATATGTTGTGGAAATGTAAAGAGCCCC<br>ATTATCTTAGCCTAAAAAAACCTTCTCTTTGGAACTTTCAGTAATACGCTTAAC<br>TGCTCATTGCTATATTGAAGTACGGATTAGAAGCCGCCGAGCGGGTGACAGCC<br>CTCCGAAGGAAGACTCTCCTCCGTGCGTCCTCGTCTTCACCGGTCGCGTTCCT<br>GAAACGCAGATGTGCCTCGCGCCGCACTGCTCCGAACAATAAAGATTCTACA<br>ATACTAGCTTTTATGGTTATGAAGAGGAAAAATTGGCAGTAACCTGGCCCCAC<br>AAACCTTCAAATGAACGAATCAAATTAACAACCATAGGATGATAATGCGATTA<br>GTTTTTTAGCCTTATTTCTGGGGTAATTAATCAGCGAAGCGATGATTTTTGATCT<br>ATTAACAGATATATAAATGCAAAAACTGCATAACCACTTTAACTAATACTTTCA<br>ACATTTTCGGTTTGTATTACTTCTTATTCAAATGTAATAAAAGTATCAACAAAAA<br>ATTGTTAATATA CCTCTATACTTTAACGTCAAGGAGAAAAAACtata
pCUP1	CTAGTTAGAAAAAGACATTTTTGCTGTCAGTCACTGTCAAGAGATTCTTTTGC<br>TGGCATTTCTTCTAGAAGCAAAAAGAGCGATGCGTCTTTTCCGCTGAACCGTT<br>CCAGCAAAAAAGACTACCAACGCAATATGGATTGTCAGAATCATATAAAAGAG<br>AAGCAAATAACTCCTTGTCTTGTATCAATTGCATTATAATATCTTCTTGTTAGTG<br>CAATATCATATAGAAGTCATCGAAATAGATATTAAGAAAAACAAACTGTACAAT<br>CAATCAATCAATCATCACATAAA
pMET25	CTTCGGATGCAAGGGTTCGAATCCCTTAGCTCTCATTATTTTTTGCTTTTTCTCT<br>TGAGGTCACATGATCGCAAAATGGCAAATGGCACGTGAAGCTGTCGATATTGG<br>GGAACTGTGGTGGTTGGCAAATGACTAATTAAGTTAGTCAAGGCGCCATCCTC<br>ATGAAAACTGTGTAACATAATAACCGAAGTGTCGAAAAGGTGGCACCTTGTCC<br>AATTGAACACGCTCGATGAAAAAAATAAGATATATATAAGGTTAAGTAAAGCG<br>TCTGTTAGAAAGGAAGTTTTTCCTTTTTCTTGCTCTCTTGTCTTTTCATCTACTA<br>TTTCCTTCGTGTAATACAGGGTCGTCAGATACATAGATACAATTCTATTACCCCC<br>ATCCATAC
lex2Op	TGCTGTATATACTCACAGCATAACTGTATATACACCCAGGG
lex2Op-pCYC1min	CAGATCCGCCAGGCGTGTATATATAGCGTGGATGGCCAGGCAACTTTAGTGCT

	GACACATATGCTGTATATACTCACAGCATAACTGTATATACACCCAGGGCAGGC<br>ATATATATATGTGTGCGACGACACATGATCATATGGCATGCATGTGCTCTGTATG<br>TATATAAAACTCTTGTTTTCTTCTTTTCTCTAAATATTCTTTCCTTATACATTAGG<br>ACCTTTGCAGCATAAATTACTATACTTCTATAGACACACAAACACAAATACACA<br>CACTAAATTAATA
3xlex2Op-pCYC1min	CAGATCCGCCAGGCGTGTATATATAGCGTGGTGCTGTATATACTCACAGCATAA<br>CTGTATATACACCCAGGGATGGCCAGGCAACTTTGCTGTATATACTCACAGCAT<br>AACTGTATATACACCCAGGGTAGTGCTGACACATATGCTGTATATACTCACAGC<br>ATAACTGTATATACACCCAGGGCAGGCATATATATATGTGTGCGACGACACATG<br>ATCATATGGCATGCATGTGCTCTGTATGTATATAAAACTCTTGTTTTCTTCTTTTC<br>TCTAAATATTCTTTCCTTATACATTAGGACCTTTGCAGCATAAATTACTATACTTC<br>TATAGACACACAAACACAAATACACACACTAAATTAATA
DEG1t_pCYC1noTATA	AATAATATATAAACCTGTATAATATAACCTTGAAGACTATATTTCTTTTCttcTTTC<br>CTTATACATtAGGACCTTTGCAGCATAAATTACTATACTTCTATAGACACACAAA<br>CACAAATACACA CACTAAATTAATA
pGPD	cagttcgagtttatcattatcaatactgccatttcaaagaatacgtaaataattaatagtagtgattttcctaactttatttagtcaaaaaa<br>ttagccttttaattctgctgtaacccgtacatgcccaaaatagggggcgggttacacagaatatataacatcgtaggtgtctgggtg<br>aacagtttattcctggcatccactaaatataatggagcccgctttttaagctggcatccagaaaaaaaaagaatcccagcaccaaa<br>atattgttttcttcaccaaccatcagttcataggtccattctcttagcgcaactacagagaacaggggcacaaacaggcaaaaaac<br>gggcacaacctcaatggagtgatgcaacctgcctggagtaaatgatgacacaaggcaattgacccacgcatgtatctatctcatt<br>ttcttacaccttctattaccttctgctctctctgatttggaaaaagctgaaaaaaaaggttgaaaccagttccctgaaattattcccta<br>cttgactaataagtatataaagacggtaggtattgattgtaattctgtaaatctatttcttaaacttcttaaattctacttttatagttagtct<br>tttttttagttttaaaacaccaagaacttagtttcgaataaacacacataaacaaacaaa
UAS(GPD-40)-150nt-(pSV40)-pCYC1min-lexOpR	GTAGGTGTCTGGGTGAACAGTTTATTCCTGGCATCCACTATGCATCTCAATTAG<br>TCAGCAACCATAGTCCCGCCCCTAACTCCGCCCATCCCGCCCCTAACTCCGCC<br>CAGTTCCGCCCATTCTCCGCCCCATCGCTGACTAATTTTTTTGCAGAGGCCGA<br>GGCCGCCTCGGCCTCTGAGCTATTCCAGAAGCATGCATGTGCTCTGTATGTATA<br>TAAAACTCTTAACTGTATATACACCCAGGGGTTTCTTCTTTTCTCTAAATATTC<br>TTTCCTTATACATTAGGACCTTTGCAGCATAAATTACTATACTTCTATAGACACA<br>CAAACACAAATACACACACTAAATTAATA
pTEF2	GCTACCTATATTCCACCATAACATCAATCATGCGGTTGCTGGTGTATTTACCAAT<br>AATGTTTAATGTATATATATATATATATGGGGCCGTATACTTACATATAGTAGATGT<br>CAAGCGTAGGCGCTTCCCCTGCCGGCTGTGAGGGCGCCATAACCAAGGTATCT<br>ATAGACCGCCAATCAGCAAACTACCTCCGTACATTCATGTTGCACCCACACATT<br>TATACACCCAGACCGCGACAAATTACCCATAAGGTTGTTTGTGACGGCGTCGT<br>ACAAGAGAACGTGGGAACTTTTTAGGCTCACCAAAAAAGAAAGAAAAAATA<br>CGAGTTGCTGACAGAAGCCTCAAGAAAAAAAAAATTCTTCTTCGACTATGCT<br>GGAGGCAGAGATGATCGAGCCGGTAGTTAACTATATATAGCTAAATTGGTTCCA<br>TCACCTTCTTTTCTGGTGTCGCTCCTTCTAGTGCTATTTCTGGCTTTTCCTATTT<br>TTTTTTTTCCATTTTTCTTTCTCTCTTTCTAATATATAAATTCTCTTGCATTTTCTA<br>TTTTTCTCTCTATCTATTCTACTTGTTTATTCCCTTCAAGGTTTTTTTTTAAGGAG<br>TACTTGTTTTTAGAA TATACGGTCAACGAACTATAATTAACTAAAC
pSNR52	CTTTGAAAAGATAATGTATGATTATGCTTTCACTCATATTTATACAGAAACTTGA<br>TGTTTTCTTTCGAGTATATACAAGGTGATTACATGTACGTTTGAAGTACAACTC

	TAGATTTTGTAGTGCCCTCTTGGGCTAGCGGTAAAGGTGCGCATTTTTTCACAC CCTACAATGTTCTGTTCAAAAGATTTTGGTCAAACGCTGTAGAAGTGAAAGTT GGTGCGCATGTTTCGG CGTTCGAAACTTCTCCGCAGTGAAAGATAAATGATC
lexOpR	AACTGTATATACACCCAGGG
3xlexOpR_trunc_pCYC1core	CGAGCAGATCCGCCAGGCGTGGCCACATAACTGTATATACACCCAGGGAAAA AAAAGCGTGGATGGCCGCCACATAACTGTATATACACCCAGGGAGGCAACTTT AGTGCTGACAGCCACATAACTGTATATACACCCAGGGCGACACATGATCATATG GCATGCATGTGCTCTGTATGTATATAAAACTCTTGTTTTCTTCTTTTCTCTAAATA TTCTTTCCTTATACATtAGGACCTTTGCAGCATAAATTA
5xlexOpR-pCYC1min	CGAGCAGATCCGCCAGGCGTGTTTACATAACTGTATATACACCCAGGGCAAAA AAAAAAGCGTGGATGGCCTTTACATAACTGTATATACACCCAGGGCAAAAGGC AACTTTAGTGCTGACATTTACATAACTGTATATACACCCAGGGCAAAAAAAAA AGCGTGGATGGCCTTTACATAACTGTATATACACCCAGGGCAAAAGGCAACTT TAGTGCTGACATTTACATAACTGTATATACACCCAGGGCAAAGACACATGATCA TATGGCATGCATGTGCTCTGTATGTATATAAAACTCTTGTTTTCTTCTTTTCTCTA AATATTCTTTCCTTATACATTAGGACCTTTGCAGCATAAATTACTATACTTCTATA GACACACAAACACAAATACACACACTAAATTAATA
pTEF1	ATAGCTTCAAAATGTTTCTACTCCTTTTTTACTCTTCCAGATTTTCTCGGACTCC GCGCATCGCCGTACCACTTCAAAACACCCAAGCACAGCATACTAAATTTCCCC TCTTTCTTCCTCTAGGGTGTCGTTAATTACCCGTACTAAAGGTTTGGAAAAGAA AAAAGAGACCGCCTCGTTTCTTTTTCTTCGTCGAAAAAGGCAATAAAAATTTT TATCACGTTTCTTTTTCTTGAAAATTTTTTTTTTGATTTTTTTCTCTTTCGATGAC CTCCCATTGATATTTAAGTTAATAAACGGTCTTCAATTTCTCAAGTTTCAGTT TCATTTTTCTTGTTCTATTACAACTTTTTTTACTTCTTGCTCATTAGAAAGAAAG CATAGCAATCTAATCTAAGTTT
tc1	CGCCTAAAACATACCAGATCGCCACCCGCGCTTTAATCTGGAGAGGTGAAGA ATACGACCACCTAGGCGAAA
tc2	GCCAATTATCTACTTAAGAACCGGTAAAACATACCAGATCGCCACCCGCGCTT TAATCTGGAGAGGTGAAGAATACGACCACCTACCGGTCAACAACAACAACAA CAACAACAACAACTCGAGGCCTAAAACATACCAGATCGCCACCCGCGCTTTA ATCTGGAGAGGTGAAGAATACGACCACCTAGGCCTCGAGAACATATGGCTAGC AAAGGAGAA
LexA-HBD(hER)	ATGAAAGCGTTAACGGCCAGGCAACAAGAGGTGTTTGATCTCATCCGTGATCA CATCAGCCAGACAGGTATGCCGCCGACGCGTGCGGAAATCGCGCAGCGTTTG GGGTTCCGTTCCCCAAACGCGGCTGAAGAACATCTGAAGGCGCTGGCACGCA AAGGCGTTATTGAAATTGTTTCCGGCGCATCACGCGGGATTCGTCTGTTGCAG GAAGAGGAAGAAGGGTTGCCGCTGGTAGGTCGTGTGGCTGCCGGTGAACCA CTTCTGGCGCAACAGCATATTGAAGGTCATTATCAGGTCGATCCTTCCTTATTC AAGCCGAATGCTGATTTCCTGCTGCGCGTCAGCGGGATGTCGATGAAAGATAT CGGCATTATGGATGGTGACTTGCTGGCAGTGCATAAAACTCAGGATGTACGTA ACGGTCAGGTCGTTGTCGCACGTATTGATGACGAAGTTACCGTTAAGCGCCTG AAAAAACAGGGCAATAAAGTCGAACTGTTGCCAGAAAATAGCGAGTTTAAAC CAATTGTCGTTGACCTTCGTCAGCAGAGCTTCACCATTGAAGGGCTGGCGGTT GGGGTTATTCGCAACGGCGACTGGCTGTCATCTGCTGGAGACATGAGAGCTG

	CCAACCTTTGGCCAAGCCCGCTCATGATCAAACGCTCTAAGAAGAACAGCCT
	GGCCTTGTCCCTGACGGCCGACCAGATGGTCAGTGCCTTGTTGGATGCTGAGC
	CCCCCATACTCTATTCCGAGTATGATCCTACCAGACCCTTCAGTGAAGCTTCGA
	TGATGGGCTTACTGACCAACCTGGCAGACAGGGAGCTGGTTCACATGATCAA
	CTGGGCGAAGAGGGTGCCAGGCTTTGTGGATTTGACCCTCCATGATCAGGTCC
	ACCTTCTAGAATGTGCCTGGCTAGAGATCCTGATGATTGGTCTCGTCTGGCGCT
	CCATGGAGCACCCAGTGAAGCTACTGTTTGCTCCTAACTTGCTCTTGGACAGG
	AACCAGGGAAAATGTGTAGAGGGCATGGTGGAGATCTTCGACATGCTGCTGG
	CTACATCATCTCGGTTCCGCATGATGAATCTGCAGGGAGAGGAGTTTGTGTGC
	CTCAAATCTATTATTTTGCTTAATTCTGGAGTGTACACATTTCTGTCCAGCACCC
	TGAAGTCTCTGGAAGAGAAGGACCATATCCACCGAGTCCTGGACAAGATCAC
	AGACACTTTGATCCACCTGATGGCCAAGGCAGGCCTGACCCTGCAGCAGCAG
	CACCAGCGGCTGGCCCAGCTCCTCCTCATCCTCTCCCACATCAGGCACATGAG
	TAACAAAGGCATGGAGCATCTGTACAGCATGAAGTGCAAGAACGTGGTGCCC
	CTCTATGACCTGCTGCTGGAGATGCTGGACGCCCACCGCCTACATGCGCCCAC
	TAGCCGTGGAGGGGCATCCGTGGAGGAGACGGACCAAAGCCACTTGGCCACT
	GCGGGCTCTACTTCATCGTAA
NLS	CCAAAGAAGAAGAGAAAAGTT
GS	GGCTCC
HIS_tag	AGCCATCATCATCATCATCACAGC
Cas12c	ACTAAACATTCTATTCCATTGCATGCTTTTAGAAATTCTGGTGCTGATGCTAGA
	AAATGGAAAGGTAGAATTGCTTTGTTGGCTAAAAGAGGTAAAGAAACTATGA
	GAACTTTGCAATTTCCATTGGAAATGTCTGAACCTGAAGCTGCAGCTATTAATA
	CTACTCCATTTGCTGTTGCTTATAATGCTATTGAAGGTACTGGAAAGGGTACTT
	TGTTTGATTATTGGGCTAAATTGCATTTGGCTGGTTTTAGATTTTTTCCATCTGG
	TGGTGCTGCTACTATTTTTAGACAACAAGCTGTTTTTGAAGATGCTTCTTGGAA
	TGCTGCTTTTTGTCAACAATCTGGTAAAGATTGGCCATGGTTGGTTCCATCTAA
	ATTGTATGAAAGATTTACTAAAGCTCCAAGAGAGGTTGCAAAAAAAGACGGT
	TCTAAAAAATCAATTGAATTTACACAAGAAAATGTTGCTAATGAATCTCATGTT
	TCTTTGGTTGGAGCTTCTATTACTGATAAAACTCCTGAAGATCAAAAAGAATTT
	TTTTTGAAAATGGCTGGTGCTTTGGCTGAAAAATTCGACTCTTGGAAATCTGC
	TAATGAAGATAGAATTGTTGCTATGAAGGTTATTGATGAATTTTTGAAATCTGA
	AGGTTTGCATTTGCCATCTTTGGAAAATATTGCTGTTAAATGTTCTGTTGAGAC
	AAAACCTGACAACGCTACTGTTGCTTGGCATGATGCTCCAATGTCTGGTGTTC
	AAAATTTGGCTATTGGTGTCTTTGCTACTTGTGCTTCAAGAATTGATAATATTTA
	TGACTTGAACGGTGGTAAATTGTCTAAATTGATTCAAGAATCTGCTACTACTCC
	AAATGTTACTGCTTTGTCTTGGTTATTTGGAAAGGGTTTGGAATATTTCAGAAC
	AACAGATATTGATACTATTATGCAAGATTTTAATATTCCTGCTTCTGCTAAAGAA
	TCTATTAAACCATTAGTTGAATCTGCTCAAGCTATTCCAACTATGACTGTTTTGG
	GTAAAAAAAATTATGCTCCATTTAGACCAAATTTTGGTGGTAAAATTGACTCTT
	GGATCGCAAATTATGCTTCAAGATTGATGTTGTTGAATGATATTTTGGAACAAA
	TTGAACCTGGTTTTGAATTGCCACAAGCTTTGTTGGATAATGAAACTTTGATGT
	CTGGTATTGATATGACTGGTGATGAATTGAAAGAATTGATTGAAGCTGTTTATG
	CTTGGGTTGATGCTGCTAAACAAGGATTGGCAACTTTGTTGGGTAGAGGTGGT

	AATGTTGATGATGCTGTTCAAACTTTTGAACAATTTTCTGCTATGATGGATACTT<br>TGAATGGTACTTTGAATACTATTTCTGCTAGATATGTTAGAGCTGTTGAAATGG<br>CTGGTAAAGACGAAGCTAGATTGGAGAAATTGATTGAATGCAAATTTGATATT<br>CCAAAGTGGTGTAAATCTGTTCCAAAATTAGTTGGTATTTCTGGTGGTTTACCA<br>AAAGTCGAAGAGGAAATTAAAGTTATGAACGCTGCATTTATGGATGTTAGAGC<br>TAGAATGTTTGTTAGATTTGAAGAAATTGCTGCTTATGTTGCTTCTAAAGGTGC<br>TGGTATGGATGTTTATGATGCTTTGGAAAAAAGAGAATTGGAACAATTGAAAA<br>AGTTGAAATCTGCAGTTCCTGAAAGGGCTCATATTCAAGCTTATAGAGCTGTTT<br>TGCATAGAATTGGAAGAGCAGTTCAAAACTGTTCTGAAAAAACTAAACAATT<br>GTTTTCTTCAAAAGTTATCGAGATGGGAGTCTTCAAGAATCCATCTCATTTAAA<br>TAATTTTATTTTTAATCAAAAAGGTGCTATTTATAGATCTCCATTTGATAGATCA<br>AGACATGCTCCATATCAATTGCATGCTGATAAATTGTTGAAAAATGACTGGATG<br>GAATTGTTGGCTGAAATTTCTACTACTTTGATGGCTTCTGAATCTACTGAACAA<br>ATGGAAGATGCTTTGAGATTGGAAAGAACTAGATTGCAATTGCAATTATCTGG<br>TTTGCCTGATTGGGAATATCCTGCTTCTTTGGCTAAACCTGATATTGAGGTCGA<br>AATTCAAACTGCTTTGAAAATGCAATTGGCTAAAGACACTGTCACTTCTGACG<br>TCTTGCAAAGGACATTTAACTTATATTCTTCTGTTTTGTCTGGTTTGACTTTTAA<br>ATTGTTGAGAAGATCTTTTTCTTTGAAGATGAGATTTTCTGTTGCTGATACTAC<br>TCAATTGATTTATGTTCCAAAAGATTGTGATTGGGCTATTCCAAAACAATATTT<br>GCAAGCTGAAGGTGAAATTGGTATTGCTGCTAGAGTTGTTACTGAATCTTCTC<br>CTGCTAAAATGGTTACTGAAGTCGAGATGAAAGAACCAAAAGCTTTGGGTCA<br>TTTTATGCAACAAGCTCCTCATGATTGGTATTTTGATGCTTCTTTGGGTGGTACT<br>CAAGTTGCTGGTAGAATCGTTGAAAAAGGTAAGGAAGTTGGTAAAGAAAGAA<br>AATTGGTTGGTTATAGGATGAGAGGTAATTCTGCTTATAAAACTGTTTTGGATA<br>AATCTTTGGTTGGAAATACTGAGTTGTCTCAATGTTCTATGATTATTGAAATTCC<br>ATATACTCAAACTGTTGATGCTGATTTTAGGGCTCAAGTTCAAGCTGGTTTGCC<br>AAAAGTCTCTATTAATTTGCCTGTTAAAGAGACTATTACTGCATCTAATAAAGA<br>TGAACAAATGTTGTTTGATAGATTTGTTGCTATTGATTTGGGTGAAAGAGGTTT<br>GGGTTATGCTGTTTTTGATGCTAAAACTTTGGAATTGCAAGAATCTGGACATAG<br>ACCAATTAAAGCTATTACTAATTTGTTAAATAGAACACATCATTATGAGCAAAG<br>ACCAAATCAGAGGCAGAAATTTCAAGCTAAATTTAATGTTAATTTGTCTGAATT<br>GAGAGAAAATACTGTTGGTGACGTTTGTCATCAGATCAATAGAATTTGTGCAT<br>ATTACAATGCTTTTCCTGTTTTGGAATATATGGTTCCTGATAGATTGGACAAACA<br>ATTGAAATCTGTTTATGAGTCTGTTACTAATAGATATATTTGGTCTTCTACTGAT<br>GCTCATAAATCTGCTAGAGTTCAATTTTGGTTGGGTGGTGAAACTTGGGAACA<br>TCCATATTTGAAGTCTGCTAAAGATAAAAAACCTTTAGTCTTGTCTCCTGGTAG<br>AGGTGCTTCTGGTAAAGGTACATCTCAGACTTGTTCTTGTTGTGGTAGAAATC<br>CATTCGATTTGATTAAAGATATGAAACCAAGAGCTAAAATTGCTGTCGTTGATG<br>GTAAAGCTAAATTGGAGAATTCAGAATTGAAATTGTTTGAAAGGAATAGAGAA<br>TCTAAAGATGATATGTTGGCTAGAAGACATAGAAATGAAAGAGCTGGTATGGA<br>ACAACCATTGACTCCTGGTAATTATACTGTTGATGAAATTAAAGCTTTGTTGAG<br>AGCTAATTTGAGAAGGGCTCCAAAAAATAGAAGAACTAAAGATACTACTGTTT<br>CTGAATATCATTGTGTTTTTTCTGATTGTGGTAAAACTATGCATGCTGATGAAA<br>ATGCTGCTGTTAATATTGGAGGAAAATTTATTGCTGATATTGAAAAA

DR(cas12c)	ATACCACCCGTGCATTTCTGGATCAATGATCCGTACCTCAATGTCCGGGCGCGC
	AGCTAGAGCGACCTGAAATCTGAAAAGCAGGATTCAGGTTGGGTTTGAGG
spacer1	CATCATGGCTGACAAAC
spacer2	TATAAATTCTCTTGCAT
spacer3	GAAAATGCAAGAGAATT
spacer4	AACAAGTAGAATAGATA
spacer5	CATAACTGTATATACAC
spacer6	CCCTGGGTGTATATACA
DR(AsCas12a)	TAATTTCTACTCTTGTAGAT
spacer7	CATAACTGTATATACACCCA
spacer8	CATCATGGCTGACAAACAAA
denAsCas12a	ACTCAATTTGAAGGATTCACCAATCTTTATCAAGTTTCTAAAACTTT
	GAGATTTGAGTTGATTCCACAAGGTAAAACCTTGAAGCACATCCAAGAACAA
	GGTTTTATCGAAGAGGACAAAGCCAGAAACGACCACTACAAGGAACTTAAAC
	CTATTATCGACAGAATCTATAAAACCTATGCCGATCAGTGCTTGCAGCTTGTTC
	AGCTTGACTGGGAAAACCTTAGTGCCGCCATTGACTCTTATAGAAAAGAAAA
	AACCGAAGAAACTAGAAACGCCCTTATTGAGGAGCAAGCTACTTATAGAAAC
	GCCATTCACGACTACTTTATCGGAAGGACCGACAACTTGACCGATGCTATTAAT
	AAGAGGCATGCCGAAATCTACAAGGGTTTGTTCAAAGCCGAACTTTTTAATGG
	TAAAGTCCTTAAACAGCTTGGAACCGTTACCACCACCGAGCATGAGAACGCT
	TTGTTGAGATCTTTCGACAAATTTACCACCTATTTTTCTGGTTTTTATAGAAATA
	GAAAGAACGTTTTTAGTGCCGAGGACATTTCTACCGCCATTCCTCATAGAATC
	GTTCAAGACAACTTCCCAAAGTTCAAGGAAAATTGCCACATCTTCACTAGACT
	TATTACCGCCGTCCCAAGTTTGAGGGAACACTTCGAGAACGTCAAGAAAGCT
	ATTGGTATCTTCGTTAGTACCTCTATCGAGGAAGTTTTTTCTTTTCCATTTTATA
	ACCAACTTTTGACCCAGACCCAGATCGACCTTTATAATCAGCTTTTGGGTGGA
	ATCTCTAGGGAAGCCGGAACCGAAAAGATCAAGGGTTTGAACGAAGTTCTTA
	ACTTGGCTATTCAGAAGAACGACGAAACCGCTCACATCATCGCTTCTTTGCCA
	CACAGATTTATTCCTTTGTTTAAACAAATCTTGTCTGATAGGAACACTCTTTCT
	TTTATTCTTGAAGAATTTAAGTCTGACGAAGAAGTTATTCAAAGTTTCTGCAA
	GTACAAGACCCTTTTGAGGAATGAGAATGTCTTGGAGACTGCCGAGGCTTTGT
	TCAATGAGCTTAATTCTATTGACTTGACTCACATTTTTATTTCTCATAAGAAATT
	GGAAACCATCAGTAGTGCCCTTTGCGACCACTGGGACACCTTGAGGAACGCT
	CTTTATGAAAGAAGAATTTCTGAGTTGACCGGAAAGATCACTAAAAGTGCTAA
	GGAAAAAGTTCAAAGATCTTTGAAACATGAAGATATTAACTTGCAAGAAATCA
	TCTCTGCCGCCGGAAAGGAACTTTCTGAAGCTTTTAAGCAAAAGACCTCTGA
	GATCCTTTCTCATGCCCATGCTGCTCTTGACCAGCCTCTTCCTACCACCCTTAA
	GAAGCAAGAGGAGAAGGAGATCTTGAAAAGTCAATTGGATAGTTTGCTTGGA
	TTGTATCATTTGCTTGATTGGTTTGCCGTCGACGAGAGTAACGAGGTCGACCC
	AGAGTTCAGTGCTAGACTTACTGGTATTAAATTGGAGATGGAACCAAGTTTGT
	CTTTTTACAATAAAGCTAGAAACTACGCTACTAAGAAACCTTACAGTGTCGAA
	AAGTTTAAGCTTAATTTCCAAATGCCTACTTTGGCTAGGGGATGGGACGTCAAT
	AGAGAGAAGAATAACGGTGCCATCCTTTTCGTCAAGAATGGACTTTATTACTT
	GGGAATCATGCCTAAACAAAAAGGTAGGTATAAAGCCCTTAGTTTCGAGCCAA

	CCGAAAAAAACCTCTGAGGGATTTGACAAAATGTATTATGACTATTTTCCAGATG
	CCGCCAAGATGATCCCAAAATGCAGTACTCAGTTGAAGGCTGTCACCGCCCA
	CTTTCAGACTCACACCACTCCAATCTTGCTTAGTAACAACTTCATTGAACCTCT
	TGAGATTACTAAGGAAATCTATGATTTGAACAATCCAGAAAAAGAACCAAAA
	AAGTTCCAGACCGCCTACGCTAAGAAAACTGGCGATCAAAAGGGTTATAGAG
	AGGCTTTGTGCAAGTGGATCGATTTCACTAGAGATTTTCTTAGTAAGTATACTA
	AAACTACTTCTATCGACTTGTCTAGTCTTAGACCATCTAGTCAGTATAAGGATC
	TTGGAGAATATTACGCTGAACTTAACCCTCTTTTGTATCATATTTCTTTTCAAAG
	AATCGCTGAAAAAGAGATCATGGATGCCGTTGAAACCGGTAAATTGTACTTGT
	TCCAAATTTATAACAAGGACTTCGCCAAGGGTCATCACGGAAAGCCTAATCTT
	CATACCCTTTACTGGACCGGACTTTTCTCTCCAGAGAACTTGGCTAAAACTAG
	TATTAAGTTGAATGGTCAAGCTGAATTGTTCTACAGACCTAAATCTAGAATGAA
	GAGGATGGCCCATAGATTGGGAGAGAAAATGCTTAATAAAAAGTTGAAGGAC
	CAAAAGACCCCTATCCCAGATACCCTTTACCAAGAGCTTTATGATTATGTTAAT
	CATAGGCTTAGTCATGATCTTAGTGACGAAGCTAGAGCCCTTTTGCCAAACGT
	CATCACCAAGGAGGTCAGTCACGAGATCATCAAAGATAGAAGATTTACCAGTG
	ACAAATTCTTCTTTCATGTCCCTATTACCTTGAATTACCAAGCTGCCAATTCTCC
	AAGTAAGTTCAACCAGAGGGTCAACGCTTACTTGAAGGAGCATCCAGAAACC
	CCTATTATCGGAATTGCTAGAGGTGAGAGAAACCTTATTTATATCACCGTCATC
	GACAGTACTGGTAAGATCCTTGAGCAGAGATCTTTGAATACCATTCAGCAGTT
	CGATTATCAAAAGAAACTTGACAATAGAGAGAAAGAAAGGGTTGCCGCCAGA
	CAAGCTTGGTCTGTCGTCGGAACTATCAAAGACCTTAAGCAAGGTTATTTGAG
	TCAAGTTATTCATGAAATTGTTGATTTGATGATCCACTACCAAGCCGTCGTTGT
	CCTTGAAAACTTGAATTTTGGATTTAAATCTAAAAGAACTGGAATCGCCGAGA
	AGGCCGTTTACCAACAGTTCGAGAAAATGTTGATCGATAAATTGAATTGTTTG
	GTCCTTAAAGACTATCCAGCCGAGAAAGTCGGTGGTGTCCTTAACCCATACCA
	ACTTACCGACCAGTTCACCTCTTTCGCCAAAATGGGAACCCAGTCTGGTTTC
	TTGTTCTATGTCCCAGCTCCATACACCTCTAAGATCGACCCACTTACCGGTTTC
	GTCGACCCTTTTGTTTGGAAAACCATCAAAAATCACGAGAGTAGAAAACATTT
	CCTTGAAGGATTCGATTTCTTGCATTATGACGTCAAAACCGGAGACTTTATTTT
	GCACTTTAAGATGAATAGAAACTTGTCTTTCCAGAGGGGTTTGCCCGGTTTCA
	TGCCAGCTTGGGACATTGTCTTTGAAAAGAATGAGACTCAGTTTGATGCCAAG
	GGAACCCCATTCATCGCTGGTAAGAGGATCGTCCCAGTTATCGAGAACCATAG
	GTTCACTGGTAGGTATAGGGACCTTTACCCAGCTAACGAGCTTATCGCCTTGCT
	TGAGGAGAAGGGAATCGTCTTTAGGGATGGTTCTAACATTCTTCCTAAATTGTT
	GGAGAACGACGACTCTCACGCTATCGATACCATGGTTGCTTTGATCAGATCTG
	TCTTGCAGATGAGGAATTCTAATGCCGCCACCGGTGAGGACTATATTAACAGT
	CCAGTCAGAGACTTGAACGGTGTCTGCTTCGACAGTAGGTTCCAGAACCCAG
	AGTGGCCAATGGATGCCGATGCCAACGGAGCTTATCACATCGCCCTTAAGGGA
	CAATTGTTGTTGAATCACCTTAAGGAAAGTAAAGATCTTAAGCTTCAGAATGG
	AATCAGTAACCAAGATTGGCTTGCCTATAT CCAAGAATTGAGAAAT
linker-VPR	TCTCCAGGTATCAGAAGATTGGACGCTTTGATCTCTACTTCTTTGTACAAGAAG
	GCTGGTTACAAGGAAGCTTCTGGTTCTGGTAGAGCTGACGCTTTGGACGACTT
	CGACTTGGACATGTTGGGTTCTGACGCTTTGGACGACTTCGACTTGGACATGT

	TGGGTTCTGACGCTTTGGACGACTTCGACTTGGACATGTTGGGTTCTGACGCT<br>TTGGACGACTTCGACTTGGACATGTTGATCAACTCTAGATCTTCTGGTTCTCCA<br>AAGAAGAAGAGAAAGGTTGGTTCTCAATACTTGCCAGACACTGACGACAGAC<br>ACAGAATCGAAGAAAAGAGAAAGAGAACTTACGAAACTTTCAAGTCTATCAT<br>GAAGAAGTCTCCATTCTCTGGTCCAACTGACCCAAGACCACCACCAAGAAGA<br>ATCGCTGTTCCATCTAGATCTTCTGCTTCTGTTCCAAAGCCAGCTCCACAACCA<br>TACCCATTCACTTCTTCTTTGTCTACTATCAACTACGACGAATTCCCAACTATGG<br>TTTTCCCATCTGGTCAAATCTCTCAAGCTTCTGCTTTGGCTCCAGCTCCACCAC<br>AAGTTTTGCCACAAGCTCCAGCTCCAGCTCCAGCTCCAGCTATGGTTTCTGCT<br>TTGGCTCAAGCTCCAGCTCCAGTTCCAGTTTTGGCTCCAGGTCCACCACAAGC<br>TGTTGCTCCACCAGCTCCAAAGCCAACTCAAGCTGGTGAAGGTACTTTGTCTG<br>AAGCTTTGTTGCAATTGCAATTCGACGACGAAGATTTGGGTGCTTTGTTGGGT<br>AACTCTACTGACCCAGCTGTTTTCACTGACTTGGCTTCTGTTGACAACTCTGA<br>ATTCCAAC AATTGTTGAACCAAGGTATCCCAGTTGCTCCACACACTACTGAAC<br>CAATGTTGATGGAATACCCAGAAGCTATCACTAGATTGGTTACTGGTGCTCAA<br>AGACCACCAGACCCAGCTCCAGCTCCATTGGGTGCTCCAGGTTTGCCAAACG<br>GTTTGTTGTCTGGTGACGAAGATTTCTCTTCTATCGCTGACATGGACTTCTCTG<br>CTTTGTTGGGTTCTGGTTCTGGTTCTAGAGACTCTAGAGAAGGTATGTTCTTGC<br>CAAAGCCAGAAGCTGGTTCTGCTATCTCTGACGTTTTCGAAGGTAGAGAAGTT<br>TGTCAACCAAAGAGAATCAGACCATTCCACCCACCAGGTTCTCCATGGGCTAA<br>CAGACCATTGCCAGCTTCTTTGGCTCCAACTCCAACTGGTCCAGTTCACGAAC<br>CAGTTGGTTCTTTGACTCCAGCTCCAGTTCCACAACCATTGGACCCAGCTCCA<br>GCTGTTACTCCAGAAGCTTCTCACTTGTTGGAAGATCCAGACGAAGAAACTTC<br>TCAAGCTGTTAAGGCTTTGAGAGAAATGGCTGACACTGTTATCCCACAAAAG<br>GAAGAAGCTGCTATCTGTGGTCAAATGGACTTGTCTCACCCACCACCAAGAG<br>GTCACTTGGACGAATTGACTACTACTTTGGAATCTATGACTGAAGATTTGAACT<br>TGGACTCTCCATTGACTCCAGAATTGAACGAAATCTTGGACACTTTCTTGAAC<br>GACGAATGTTTGTTGCACGCTATGCACATCTCTACTGGTTTGTCTATCTTCGAC<br>ACTTCTTTGTTC
Mxi1	ATGATTAATGTGCAAAGGCTGTTAGAAGCCGCAGAGTTTTTAGAAAGAAGAG<br>AAAGAGAATGCGAACACGGGTATGCCAGTTCTTTCCCTAGCATGCCCTCTCCC<br>AGATGA
B42	ATCAATAAAGATATCGAGGAGTGCAATGCCATCATTGAGCAGTTTATCGACTAC<br>CTGCGCACCGGACAGGAGATGCCGATGGAAATGGCGGATCAGGCGATTAACG<br>TGGTGCCGGGCATGACGCCGAAAACCATTCTTCACGCCGGGCCGCCGATCCA<br>GCCTGACTGGCTGAAATCGAATGGTTTTCATGAAATTGAAGCGGATGTTAACG<br>ATACCAGCCTCTTGCT GAGTGGAGAT
VP64	GACGCTTTGGACGACTTCGACTTGGATATGCTGGGTTCTGATGCGCTAGATGA<br>CTTTGACCTCGACATGCTTGGAAGTGACGCCTTAGATGATTTTGACCTGGATAT<br>GCTTGGA TCAGACGCTCTGGACGATTTCGACTTAGACATGCTT

AcrVA1	TATGAAGCTAAAGAAAGATATGCTAAAAAAAAAATGCAAGAAAACACCAAG ATCGACACCCTTACCGACGAACAGCATGATGCCTTGGCTCAGTTGTGCGCCT TTAGACACAAGTTCCACTCTAACAAGGATAGTTTGTTTTTGTCTGAATCTGCT TTCTCTGGTGAATTTTCTTTTGAAATGCAATCTGACGAGAACAGTAAGTTGAG GGAGGTCGGTTTGCCAACCATCGAGTGGAGTTTCTACGACAACTCTCATATT CCAGATGATTCTTTTAGAGAATGGTTTAATTTCGCTAACTACTCTGAGCTTTC TGAAACCATCCAAGAACAAGGTCTTGAATTGGACTTGGACGATGACGAAACC TACGAGCTTGTCTACGACGAATTGTACACCGAGGCCATGGGTGAATACGAGG AGTTGAACCAAGATATTGAGAAGTATTTGAGGAGGATCGACGAGGAGCATG GTACCCAGTACTGCCCAACCGGTTTTG CCAGATTGAGA
AcrVA4	TATGAAGCTAAAGAAAGATATGCTAAAAAAAAAATGCAAGAAAACACCAAG ATCGACACCCTTACCGACGAACAGCATGATGCCTTGGCTCAGTTGTGCGCCT TTAGACACAAGTTCCACTCTAACAAGGATAGTTTGTTTTTGTCTGAATCTGCT TTCTCTGGTGAATTTTCTTTTGAAATGCAATCTGACGAGAACAGTAAGTTGAG GGAGGTCGGTTTGCCAACCATCGAGTGGAGTTTCTACGACAACTCTCATATT CCAGATGATTCTTTTAGAGAATGGTTTAATTTCGCTAACTACTCTGAGCTTTC TGAAACCATCCAAGAACAAGGTCTTGAATTGGACTTGGACGATGACGAA ACCTACGAGCTTGTCTACGACGAATTGTACACCGAGGCCATGGGTGAATACG AGGAGTTGAACCAAGATATTGAGAAGTATTTGAGGAGGATCGACGAGGAGC ATGGTACCCAGTACTGCCCAACCGGTTTTG CCAGATTGAGA
AcrVA5	AAGATCGAATTGTCTGGTGGTTACATCTGTTACTCTATCGAAGAAGACGAAG TTACTATCGACATGGTTGAAGTTACTACTAAGAGACAAGGTATCGGTTCTCA ATTGATCGACATGGTTAAGGACGTTGCTAGAGAAGTTGGTTTGCCAATCGGT TTGTACGCTTACCCACAAGACGACTCTATCTCTCAAGAAGACTTGATCGAATT CTACTTCTCTAACGACTTCGAATACGACCCAGACGACGTTGACGGTAGATTG ATGAGATGGT CT
GFP	GAACTTTTCACTGGAGTTGTCCCAATTCTTGTTGAATTAGATGGTGATGTTAA TGGGCACAAATTTTCTGTCAGTGGAGAGGGTGAAGGTGATGCTACATACGGA AAGCTTACCCTTAAATTTATTTGCACTACTGGAAAACTACCTGTTCCATGGCC AACACTTGTCACTACTTTGACCTATGGTGTTCAATGCTTTTCCCGTTATCCGG ATCATATGAAACGGCATGACTTTTTCAAGAGTGCCATGCCCGAAGGTTATGT ACAGGAACGCACTATATCTTTCAAAGATGACGGGAACTACAAGACGCGTGCT GAAGTCAAGTTTGAAGGTGATACCCTTGTTAATCGTATCGAGTTAAAAGGTA TTGATTTTAAAGAAGATGGAAACATTCTCGGACACAAACTCGAGTACAACTA TAACTCACACAATGTATACATCACGGCAGACAAACAAAAGAATGGAATCAA AGCTAACTTCAAAATTCGCCACAACATTGAAGATGGATCCGTTCAACTAGCA GACCATTATCAACAAAATACTCCAATTGGCGATGGCCCTGTCCTTTTACCAG ACAACCATTACCTGTCGACACAATCTGCCCTTTCGAAAGATCCCAACGAAAA GCGTGACCACATGGTCCTTCTTGAGTTTGTAACTGCTGCTGGGATTACACATG GCATGGATGAGCTCTACAAATAA

yEGFP	ATGTCTAAAGGTGAAGAATTATTCACTGGTGTTGTCCCAATTTTGGTTGAATT
	AGATGGTGATGTTAATGGTCACAAATTTTCTGTCTCCGGTGAAGGTGAAGGT
	GATGCTACTTACGGTAAATTGACCTTAAAATTTATTTGTACTACTGGTAAATT
	GCCAGTTCCATGGCCAACCTTAGTCACTACTTTCGGTTATGGTGTTCAATGTT
	TTGCGAGATACCCAGATCATATGAAACAACATGACTTTTTCAAGTCTGCCAT
	GCCAGAAGGTTATGTTCAAGAAAGAACTATTTTTTTCAAAGATGACGGTAAC
	TACAAGACCAGAGCTGAAGTCAAGTTTGAAGGTGATACCTTAGTTAATAGAA
	TCGAATTAAAAGGTATTGATTTTAAAGAAGATGGTAACATTTTAGGTCACAA
	ATTGGAATACAACTATAACTCTCACAATGTTTACATCATGGCTGACAAACAA
	AAGAATGGTATCAAAGTTAACTTCAAAATTAGACACAACATTGAAGATGGTT
	CTGTTCAATTAGCTGACCATTATCAACAAAATACTCCAATTGGTGATGGTCCA
	GTCTTGTTACCAGACAACCATTACTTATCCACTCAATCTGCCTTATCCAAAGA
	TCCAAACGAAAAGAGgGACCACATGGTCTTGTTAGAATTTGTTACTGCTGCTG
	GTATTACCCATGGTATGGATGAATTGTACAAATAA
CYC1t	CATGTAATTAGTTATGTCACGCTTACATTCACGCCCTCCCCCCACATCCGCTC
	TAACCGAAAAGGAAGGAGTTAGACAACCTGAAGTCTAGGTCCCTATTTATTT
	TTTTATAGTTATGTTAGTATTAAGAACGTTATTTATATTTCAAATTTTTCTTTT
	TTTTCTGTACAGACGCGTGTACGCATGTAACATTATACTGAAAACCTTGCTTG
	AGAAGGTTTTGGGACGCTC GAAGGCTTTAATTTGCAAGCT
SUP4t	TTTTTTTGTTTTTTATGTCT
mTGuo1	TATATAACTGTCTAAAATAAAGAGTATCATCTAAAAA
Tsynth6	TATATATTTAATAAAGAGTATCATCTTTCAAA
Tsynth24	TGGGTGGTATGTTATATAACTGTCTAGAAATAAAGAGTATCATCTTTCAAA
